# *N*-Sulfonyl amidine polypeptides: new polymeric biomaterials with conformation transition responsive to tumor acidity†

**DOI:** 10.1039/d3sc05504c

**Published:** 2023-12-20

**Authors:** Xiang Xu, Jinjuan Ma, Aiguo Wang, Nan Zheng

**Affiliations:** a School of Chemical Engineering, Dalian University of Technology Dalian 116024 China nzheng@dlut.edu.cn; b Department of Comparative Medicine Laboratory Animal Center, Dalian Medical University Dalian 116000 China wangaiguotl@hotmail.com; c Dalian University of Technology Corporation of Changshu Research Institution Suzhou 215500 China

## Abstract

Manipulation of pH responsiveness is a frequently employed tactic in the formulation of trigger-responsive nanomaterials. It offers an avenue for “smart” designs capitalizing on distinctive pH gradients across diverse tissues and intracellular compartments. However, an overwhelming majority of documented functional groups (>80%) exhibit responsiveness solely to the heightened acidic *milieu* of intracellular pH (about 4.5–5.5). This scenario diverges markedly from the moderately acidic extracellular pH (∼6.8) characteristic of tumor microenvironments. Consequently, systems predicated upon intracellular pH responsiveness are unlikely to confer discernible advantages concerning targeted penetration and cellular uptake at tumor sites. In this study, we elucidated the extracellular pH responsiveness intrinsic to *N*-sulfonyl amidine (SAi), delineating a method to synthesize an array of SAi-bearing polypeptides (SAi-polypeptides). Notably, we demonstrated the pH-dependent modulation of SAi-polypeptide conformations, made possible by the protonation/deprotonation equilibrium of SAi in response to minute fluctuations in pH from physiological conditions to the extracellular *milieu* of tumors. This dynamic pH-triggered transition of SAi-polypeptides from negatively charged to neutrally charged side chains at the pH outside tumor cells (∼6.8) facilitated a transition from coil to helix conformations, concomitant with the induction of cellular internalization upon arrival at tumor sites. Furthermore, the progressive acidification of the intracellular environment expedited drug release, culminating in significantly enhanced site-specific chemotherapeutic efficacy compared with free-drug counterparts. The distinct pH-responsive attributes of SAi could aid the design of tumor acidity-responsive applications, thereby furnishing invaluable insights into the realm of smart material design.

## Abstract 2

The novel pH-responsive property of the *N*-sulfonyl amidine (SAi) structure was applied in the design of conformation–transition polypeptides as a “smart” drug-delivery system to achieve site-specific tumor penetration and drug release.

## Graphical abstract

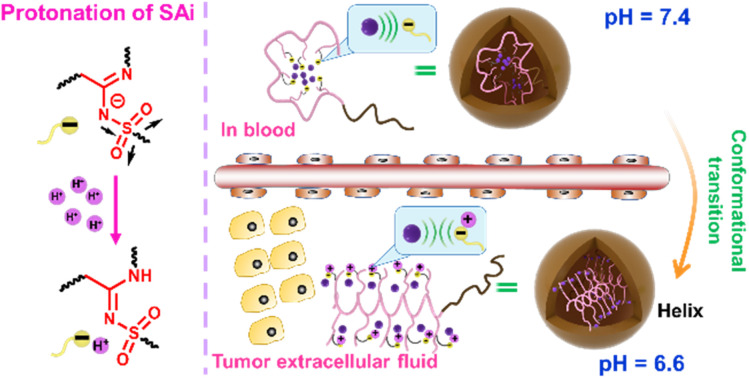

## Introduction

Stimuli-responsive biomaterials show great potential in the drug-delivery field by improving blood circulation, tumor penetration, cellular internalization, and intracellular drug release.^1–9^ Responsiveness to pH has been used widely to design trigger-responsive nanomaterials because the pH in different tissues and cellular compartments varies tremendously.^10–19^ For example, blood and most normal organs have a relatively mild basic value (∼7.4). Tumors have an acidic microenvironment with a dysregulated pH of 6.8 caused by the production and accumulation of lactic acid induced by rapid metabolization. The intracellular environment has a more acidic condition with pH of 4.5–5.5.^20–22^ Even though numerous pH-triggered drug-delivery systems have been developed, most have focused on the responsiveness to intracellular pH.^23–30^ Less attention has been paid to the development of nanocarriers that can respond sensitively to the acidic extracellular environment of the tumor because of the slight pH change (from 7.4 to 6.8), relatively weak acidity, and structure limitation.^31–34^ Currently, only maleic acid amides,^34,35^ hydrazones,^36,37^ imines,^38,39^ and sulfonamides^40–44^ have been reported with regard to responsiveness to the weak acidic environment. An intracellular pH-responsive system is an advantage of intracellular drug release but it is not beneficial for tumor site-specific penetration and cell uptake. Considering the great promise and lack of abundant optional structures, developing unknown structures or exploring the undiscovered properties of known structures that can respond sensitively to tumor acidity is challenging.

Recently, our group reported a novel method of multicomponent polymerization for the synthesis of *N*-sulfonyl amidine (SAi) polymers which, unexpectedly, showed zwitterionic properties upon pH changes.^45^ In general, it is believed that the amidine group, as a “base”, can be protonated and shows positive charges at neutral or acidic conditions, but has neutral charges at basic conditions. However, SAi shows a similar characteristic with a “weak acid” instead of a “base” due to the electron-withdrawing feature of the sulfonyl group. Such a hallmark inspires us to further explore its relationship between structure and p*K*_a_ value, and expand its potential in the design of pH-responsive polymers.

Synthetic polypeptides are a class of protein mimics with rich structural diversity, good biocompatibility, and degradability.^46–48^ The ability to form important secondary structures (*e.g.*, α-helix) endows them with unique properties and functions superior to those of unstructured polymers. Moreover, polypeptides with specific groups can also adopt different secondary structures and conformations responsive to various external or internal triggers, such as pH,^49,50^ redox,^51,52^ enzymes,^53,54^ temperature,^55^ and light.^56^ The coil-to-helix conformation transition of polypeptides has been demonstrated with the selective activation of tumor-cell penetration.^57,58^ Thus, polypeptides serve as promising candidates bearing Sai in response to tumor acidity.

**Scheme 1:**
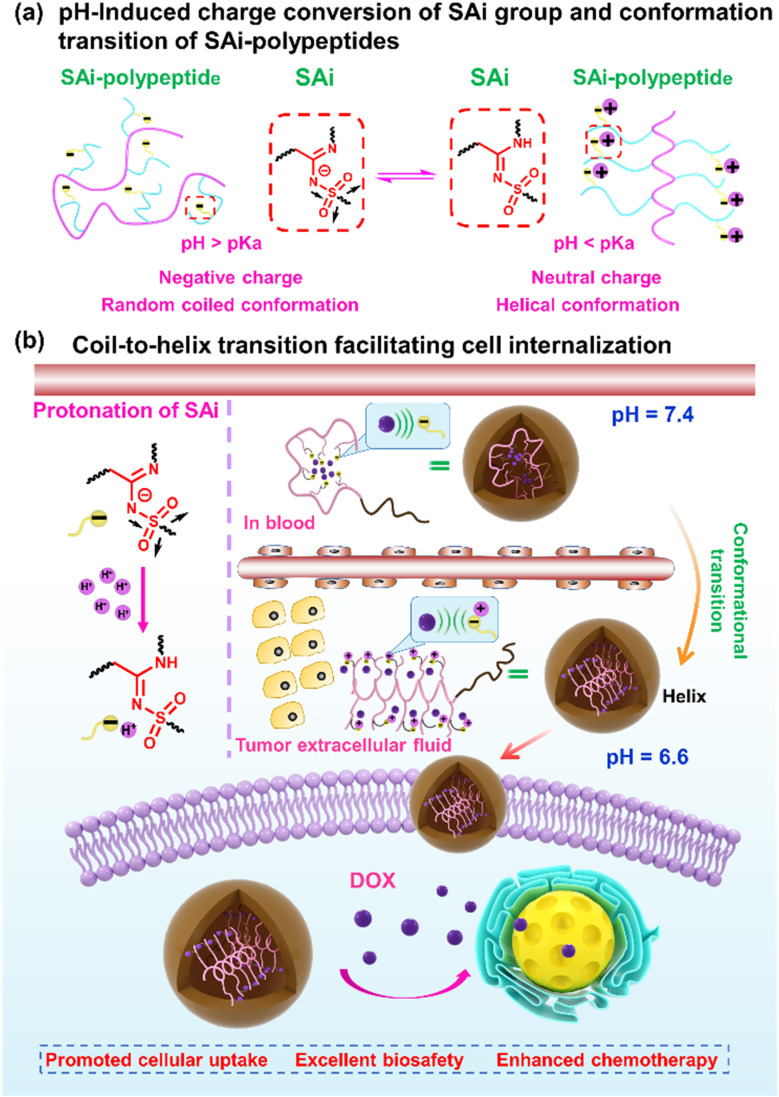
Illustration showing: (a) pH-induced charge conversion of an SAi group and conformation transition of SAi-polypeptides; (b) coil–helix conformation transition responsive to an acidic tumor microenvironment, promoting cell internalization and chemotherapy.

In this work, we systemically investigated the chemical–physical properties of the SAi group: protonation ability, p*K*_a_, and electronic features. Then, a library of SAi-polypeptides was synthesized *via* the post-polymerization modification of polypeptides using the multicomponent reaction of a terminal alkyne, sulfonyl azide, and primary/secondary amine. Unlike the strategy to install amidine along the peptide backbone,^59–61^ such a modification strategy can incorporate diverse SAi structures on the side chains of polypeptides in a facile and mild manner. Owing to the pH-induced charge conversion of the SAi group, the SAi-polypeptides exhibited a random coil-to-helix transition across a pH range from 6.2 to 7.0, which was similar to the acidic pH of the tumor. Such a transition in pH was related to the p*K*_a_ and could be finely “tuned” by the chemical structure of SAi. Upon screening, polypeptides with DP = 20 (P_20_-PA, P_20_-HA, and P_20_-DA) exhibited suitable values of p*K*_a_ and transition pH, which were selected as the top-performing materials and designed further as drug-delivery carriers. Upon circulation in the blood, SAi-polypeptides showed negative charges and a random-coil conformation, leading to tight encapsulation of the “drug payload” and deactivation of cell internalization. At tumor tissues, these SAi-polypeptides could be protonated efficiently to have a neutral charge with the conformation altered to a helix, thereby facilitating cell penetration and internalization at the tumor site. Once internalized into the cell, the continuously decreased pH triggered drug release within the cell. Finally, the desired anti-tumor efficacy was demonstrated in a mouse model. We reported, for the first time, the undiscovered pH-responsive feature of the SAi group. In this way, we provided a novel insight for the design of conformation–transition polypeptides and responsive nanomaterials (Scheme 1).

## Results and discussion

**Fig. 1:**
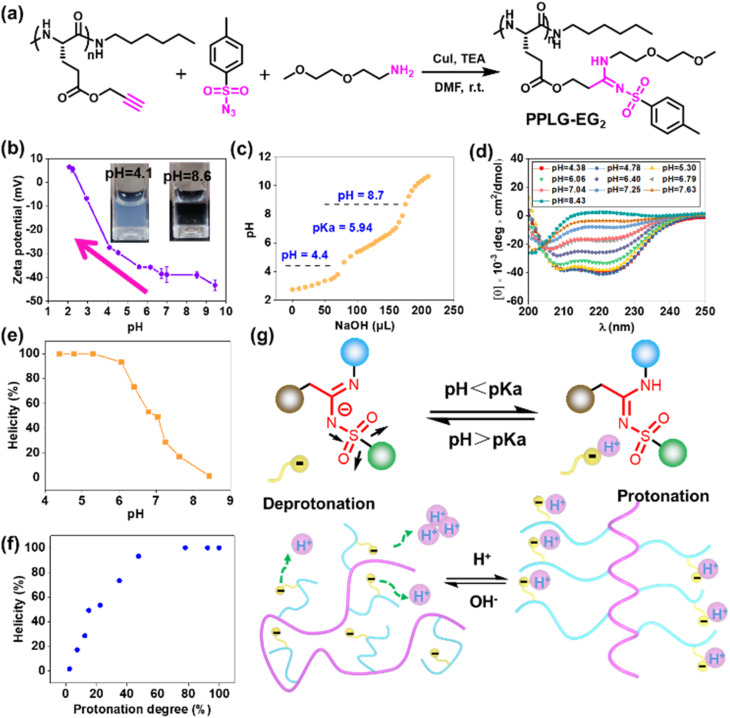
(a) Synthetic route and chemical structure of PPLG-EG_2_. (b) Zeta potential of PPLG-EG_2_ at different pH values. (c) pH titration curve of PPLG-EG_2_ adjusted using NaOH (0.1 M) solution. (d) CD spectra of PPLG-EG_2_ at different pH values. (e) pH–helicity plot of PPLG-EG_2_. (f) Protonation degree–helicity plot of PPLG-EG_2_. (g) Coil-to-helix transition caused by protonation/deprotonation of Sai (schematic). Molar ellipticities were calculated in degree × cm^2^ × dmol^−1^. Helicity was calculated using the equation: [*θ*]_*λ*_ = (MRW × *θ*_*λ*_)/(*d* × *c*), where helicity = (−[*θ*]_222_ + 3000)/39 000, MRW is the mean residue weight, *θ*_*λ*_ is the observed ellipticity at wavelength *λ* (*i.e.* 222 nm), *d* is the path length (mm), and *c* is the concentration (mg mL^−1^).

To study the pH-responsivity of polypeptides bearing SAi, we initially synthesized a water-soluble polypeptide with oligo(ethylene glycol) (EG) groups (PPLG-EG_2_). PPLG-EG_2_ was synthesized through a typical three-component reaction involving terminal alkyne-polypeptides, *p*-toluenesulfonyl azide, and 2-(2-methoxyethoxy)ethanamine (Fig. 1a, S7 and S10†). Notably, PPLG-EG_2_ exhibited a zwitterionic property with an isoelectric point (pI) around 2.6 (Fig. 1b). PPLG-EG_2_ exhibited a gradually increased negative charge with pH increasing from 2.6, but exhibited a slightly positive charge when pH < 2.6. pH titration of PPLG-EG_2_ in aqueous solution revealed p*K*_a_ ∼ 5.94, and a buffering effect was observed (Fig. 1c). Such results indicated that PPLG-EG_2_ was deprotonated by adding NaOH solution during pH titration. As shown in CD spectra, PPLG-EG_2_ displayed a pH-dependent conformational transition (Fig. 1d), indicating that the protonation/deprotonation of SAi induced conformational changes in PPLG-EG_2_. This phenomenon occurred because the deprotonation of SAi introduced negative charges on the side chains, which destabilized the helix conformation of the polypeptides. This result was in accordance with the data of a report stating that the side-chain ionic interaction destabilizes the helical structure of polypeptides.^62^ The helicity of PPLG-EG_2_ was calculated, and the correlation between helicity and pH is shown in Fig. 1e. The helicity remained >90% when pH < 6.08 but dropped markedly when pH increased from 6.06 to 8.43 due to the ionic interaction induced by deprotonation. When the protonation degree exceeded 80%, PPLG-EG_2_ displayed a high helicity of ∼100%. The quantitative results shown in Fig. 1f also revealed the correlation between the helix-to-coil transition and degree of protonation. Unlike conventional amidines or amines, SAi exhibited an intriguing neutral charge under acidic pH, which could be attributed to the weak acid properties of SAi in aqueous solution (p*K*_a_ < 7). To explore the possible deprotonation mechanism of SAi, we synthesized small molecules as model compounds. Their ^1^H NMR spectra were analyzed, and the appearance of paired peaks revealed isomerization (Fig. S21†). Similar to sulfonamides, SAi exhibited various resonance forms and showed comparable ionization behavior. The strong electron-withdrawing sulfone group facilitated the hydrogen on nitrogen being prone to dissociation, similar to that seen in sulfonamide groups (Fig. 1g). When pH < p*K*_a_, the SAi remained neutral whereas, at pH > p*K*_a_, the SAi released a proton to form an anionic group. As a control, the CD spectra of poly-l-histidine (PLH) showed a random-coil conformation at low pH (<6.00), and started to adopt a β-sheet conformation at pH = 6.00 (Fig. S22†). This was different from the transition behaviors of SAi-polypeptides, which adopted an α-helical conformation at weakly acidic pH. This conformational transition and hydrophobic variation could alter their assembly behavior. Furthermore, we characterized PPLG-EG_2_ in aqueous solutions at various pH values using DLS measurements. Interestingly, as pH decreased, the size changed from 270 nm to 170 nm, and particles tended to become stable (Fig. S23†). This feature was attributed to the improved hydrophobicity of the nanoparticle core. In addition, we observed pH-triggered solubility-transition behavior through images captured at different pH values (Fig. S24†).

**Fig. 2:**
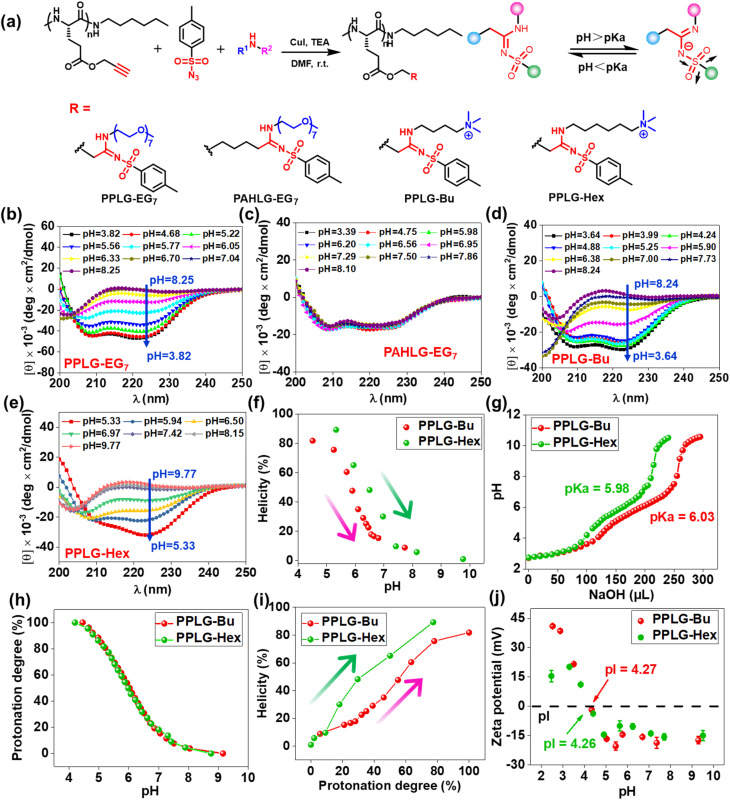
(a) Synthetic route and chemical structure of polypeptides with different SAi structure. CD spectra of (b) PPLG-EG_7_, (c) PAHLG-EG_7_, (d) PPLG-Bu, and (e) PPLG-Hex. (f) pH–helicity plots of PPLG-Bu and PPLG-Hex. (g) pH titration curves of PPLG-Bu and PPLG-Hex adjusted using NaOH (0.1 M) solution. (h) Protonation degree as a function of pH. (i) Protonation degree–helicity plots of PPLG-Bu and PPLG-Hex. (j) Zeta potential of PPLG-Bu and PPLG-Hex at different pH values.

The remarkable pH-responsive property of the SAi group inspired us to design and synthesize a series of SAi-polypeptides with different structural features to examine their pH-triggered conformational transitions (Fig. 2a, S1–S6, and S11–S14†). PPLG-EG_7_ with longer EG chains displayed a similar transition pattern to that of PPLG-EG_2_ as shown in CD spectra (Fig. 2b). To attenuate the ionic effect of SAi on conformation, PAHLG-EG_7_ was synthesized to extend the distance from the SAi to the peptide backbone. As expected, PAHLG-EG_7_ adopted a stable α-helical conformation in a broad range of pH (Fig. 2c). That result suggested the disruptive action of SAi was weakened if SAi was positioned farther from the main chain. To further support the central role of SAi, we synthesized another two types of polypeptides (PPLG-Bu and PPLG-Hex) with quaternary ammonium at the side-chain terminus. The presence of a quaternary ammonium group could provide the pH-independent water solubility and independent positive charges of polypeptides. As shown in Fig. 2d–f, PPLG-Bu and PPLG-Hex displayed a similar coil-to-helix transition with decreasing pH, suggesting that the quaternary ammonium group did not alter the pH-triggered conformation transition. Owing to increasing hydrophobicity, PPLG-Hex showed a lower peak at 208 nm compared to 222 nm at lower pH values, revealing an aggregation of the helix at the nanometric level.^63^ Additionally, at pH values below 5.3, the helix of PPLG-Hex was disrupted due to precipitation (Fig. S25†). The pH titrations were also carried out in an aqueous solution (Fig. 2g). PPLG-Bu and PPLG-Hex owned the p*K*_a_ around 5.98 and 6.03, respectively. Additionally, both of them performed similar protonation behaviors (Fig. 2h). Furthermore, we observed a positive correlation between helicity and protonation degree in these polypeptides (Fig. 2i). The zeta potential was also tested (Fig. 2j), which revealed these polypeptides to have obvious zwitterionic properties. Negative potentials were enhanced gradually with pH increase, albeit with a pH-independent ammonium group as the positive-charge domain. Due to the ammonium group, the pI values of these polypeptides were higher than that of PPLG-EG_2_. Collectively, All these results indicated that the protonation of SAi had a critical role in the conformation transition of polypeptides.

**Fig. 3:**
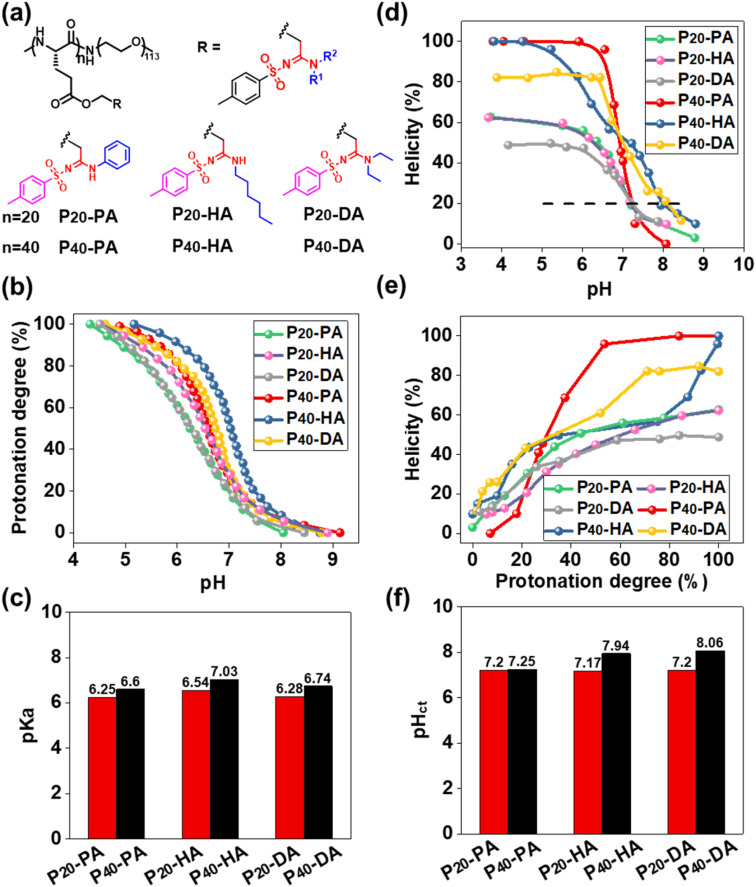
(a) Chemical structures of amphiphilic polypeptides with different SAi structures. (b) Protonation degree as a function of pH. (c) p*K*_a_ of SAi-polypeptides. (d) Helicity as a function of pH. (e) Helicity as a function of protonation degree. (f) pH_ct_ of SAi-polypeptides.

The function of pH-responsive polypeptides encouraged us to further explore their function as biomaterials. Six PEGylated polypeptides with different DP and bearing different SAi groups were prepared using PEG_5000_-NH_2_ as the initiator (Fig. 3a, S8, S9, and S15–S20†). pH titrations were performed to determine the proton-buffering capacity (Fig. S26†). The proton-buffering range for all PEGylated SAi-polypeptides was between pH 6 and pH 7. Their protonation behaviors and p*K*_a_ values are summarized in Fig. 3b and c. DP and the side-chain structures could manipulate p*K*_a_. The basicity of N-terminal substituents seemed to have a critical role in determining p*K*_a_. The basicity in aqueous solution followed the order: HA > DA > PA, which correlated with their p*K*_a_ values. SAi-polypeptides with higher DP showed increased p*K*_a_. The hydrophilic PEG length was identical, so the lower DP of polypeptides made them relatively more hydrophilic. A more hydrophilic environment makes acidic units (SAi) stronger, leading to lower p*K*_a_ values.^64^ CD of these polypeptides was also performed at different pH values (Fig. S27†). As expected, the helicity was increased with a decrease in pH and increase in protonation degree (Fig. 3d and e). When fully protonated, SAi-polypeptides with higher DP possessed higher helicities, consistent with previous results.^65,66^ As shown in Fig. S26,† polypeptides showed a random-coil conformation with helicity < 20%, and the α-helix had helicity > 20%. A critical helicity of 20% was identified as the conformational transition pH (pH_ct_). These pH_ct_ values were close to the corresponding p*K*_a_ values (within 1 unit of pH) (Fig. 3f). pH_ct_ values were important for polypeptides to modulate their conformational transition behaviors under a slightly acidic environment. Due to their pH_ct_ being <7.4, the polypeptides (SAi-polypeptides with DP = 20 and P_40_-PA) were expected to respond to the microenvironment of tumors or bacteria.

Amphiphilic copolymers containing hydrophilic and hydrophobic segments can self-assemble spontaneously in aqueous solutions. The critical micelle concentration (CMC) of P_20_-PA, P_20_-HA, and P_20_-DA nanoparticles was determined to be 16.2, 11.9, and 19.7 μg mL^−1^, respectively (Fig. S28†). To further evaluate their ability to encapsulate drugs, doxorubicin (DOX), as a typical chemotherapeutic drug, was encapsulated by P_20_-PA, P_20_-HA, and P_20_-DA through a nanoprecipitation method. The diameters of DOX-loaded nanoparticles (DOX-NPs) were around 120–180 nm as tested by DLS (Table 1, Fig. S29–S31†). The results in Table 1 indicated that these DOX-NPs exhibited relatively higher drug-loading content (DLC) and drug-loading efficiency (DLE) compared with those of most amphiphilic copolymers such as poly(ethylene glycol)-poly(lactic acid) (PEG-PLA).^67^ The DLC values of the three DOX-NPs were up to 32.6%, 31.1%, and 29.7%, with corresponding DLE values of 99%, 93.5%, and 89.2%, respectively. The high values of DLC and DLE were mainly attributed to the interactions between DOX and the SAi structure. DOX, as a weak base with p*K*_a_ of 8.3, higher than the pH of the phosphate-buffered saline (PBS; ∼7.4), likely underwent protonation to provide positive charges.^68^ Electrostatic interactions between DOX and the SAi structure could be one of the reasons for the high DLC value. Other interactions (*e.g.*, hydrogen bonding, hydrophobic) could also have contributed to the results. Isothermal titration calorimetry (ITC) provided further insights into the interaction forces, revealing an enthalpy–entropy co-driven process, with Δ*H* < 0 and −*T*Δ*S* < 0 (Fig. S32†). The negative Δ*G* value indicated that the combination occurred spontaneously. Then, we analyzed the ^1^H NMR spectra of a mixture of DOX and SAi small molecules (PA, HA, and DA). The phenolic hydroxyl group in DOX displayed a broad resonance signal and became sharp after the addition of SAi small molecules (Fig. S33†). As shown in Fig. S34a,† The UV-vis spectra had a red shift in the absorption peak after drug encapsulation, indicative of π–π stacking.^67^ The remarkable decrease in the mean fluorescence intensity (MFI) of the emission band at the same excitation wavelength and DOX concentration also demonstrated the interaction between the SAi-polypeptides and DOX (Fig. S34b†). Among them, PA@DOX-2 showed the highest DLC, which was reasonable because the PA structure had stronger hydrophobicity, typical π–π interaction, and relatively lower p*K*_a_.

**Table 1:** Summary of the parameters of drug-loaded micelles

Micelle	Polymer	Drug/polymer (w/w)	DLC^a^ (%)	DLE^b^ (%)	Size (nm)	PDI	Zeta potential (mV)
PA@DOX-1	P_20_-PA	1 : 1	34.7	69.5	172.6	0.230	−3.2
PA@DOX-2	P_20_-PA	1 : 2	32.6	99	122.1	0.278	−4.2
PA@DOX-3	P_20_-PA	1 : 5	16.2	99	139.0	0.187	−5.2
HA@DOX-1	P_20_-HA	1 : 1	31.5	63.3	142.5	0.265	−2.9
HA@DOX-2	P_20_-HA	1 : 2	31.1	93.5	121.2	0.256	−3.2
HA@DOX-3	P_20_-HA	1 : 5	13.7	97.5	181.0	0.322	−4.5
DA@DOX-1	P_20_-DA	1 : 1	31.5	64.7	159.6	0.211	−2.8
DA@DOX-2	P_20_-DA	1 : 2	29.7	89.2	122.2	0.255	−3.5
DA@DOX-3	P_20_-DA	1 : 5	15.6	93.9	135.2	0.228	−4.1

a DLC = (amount of loaded drug/amount of drug-loaded NPs) × 100%.

b DLE = (amount of loaded drug/amount of feeding drug) × 100%.

The release profiles of DOX-loaded micelles were investigated under different pH values at 37 °C to represent the acidic condition and physiological environment. As shown in Fig. S35,† the environmental acidity had a significant effect on DOX release. At pH 7.4, percent release was slower (53% within 128 h) whereas at pH 6.8, ∼67% of DOX was released. Such different release behaviors of DOX could be ascribed to the improved hydrophilicity of DOX and additional conformational transition. Around 80% of DOX was released at pH 5.5, indicating that NPs were sensitive to endo/lysosomal pH, allowing more drugs to be released intracellularly. Such pH-sensitive behavior would be advantageous in reducing drug loss during blood circulation and controlling drug release at the tumor site. Drug-releasing behavior was also observed from the fluorescent intensity. Compared with DOX·HCl, DOX-NPs showed higher MFI with increasing pH, indicating drug release from DOX-NPs (Fig. S36†).

**Fig. 4:**
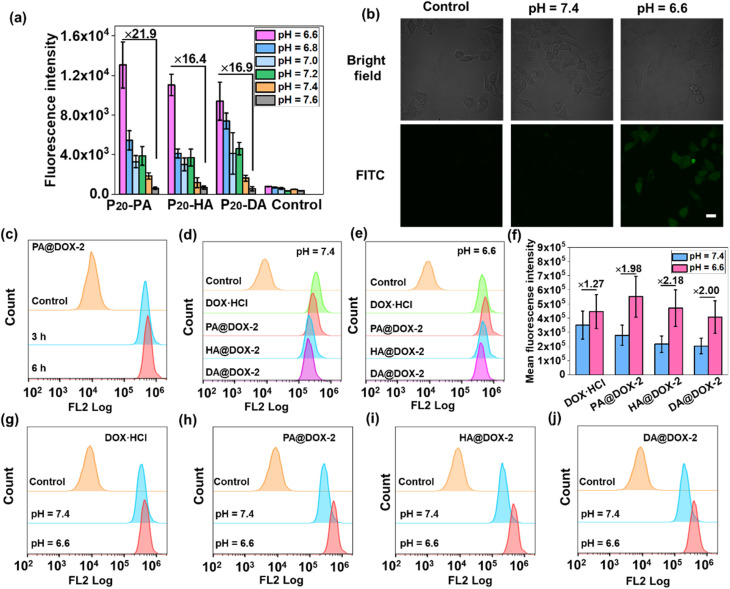
(a) Level of FITC-Tris uptake of HeLa cells following co-incubation with SAi-polypeptides at pH 6.6–7.6. Parallel experiments were carried out three times (*n* = 3). “Control” denotes cells treated without polypeptides. (b) CLSM images showing the level of FITC-Tris uptake of HeLa cells treated or not treated with P_20_-PA for 4 h at pH = 6.6 and 7.4. Scale bar = 20 μm. (c) FCM histogram profiles of HeLa cells treated with PA@DOX-2 for 3 or 6 h. FCM histogram profiles of HeLa cells treated with DOX-NPs for 6 h at pH 6.6 (d) or 7.4 (e). (f) Mean fluorescence intensity of DOX-NPs obtained from FCM analysis. Comparison of FCM histograms of DOX-NPs at pH 6.6 and 7.4: DOX·HCl (g), PA@DOX-2 (h), HA@DOX-2 (i), DA@DOX-2 (j). The DOX concentration was 35 μg mL^−1^.

We wished to ascertain whether the conformational transition behaviors of DOX-NPs would benefit their internalization by cells at the extracellular pH of a tumor. The cell-penetration ability and cell-uptake behaviors of polypeptides were investigated. FITC-Tris has been reported to be a membrane-impermeable fluorescent dye whose level of uptake could be used to evaluate cell penetration and pore formation on cell membranes.^65^ As shown in Fig. 4a, free FITC-Tris could not be taken up by cells at pH ranging from 6.6 to 7.6. After treatment with SAi-polypeptides, efficient uptake was observed from the increasing fluorescence of FITC in cells. A 20-fold increase in cell-penetration level for SAi-polypeptides when the pH changed from 7.6 to 6.6. The pH-dependent level of FITC uptake was attributed to the coil–helix transition because the helical structure (shown at pH ∼ 6.6) could induce pore formation notably. Confocal laser scanning microscopy (CLSM) was also used to determine FITC uptake. As shown in Fig. 4b, the intensity of FITC at pH 6.6 was higher than that at pH 7.4, which was related to conformational transition (helix at pH 6.6 and random coil at pH 7.4). As a control, an analogue polypeptide with a random-coil conformation (DL-P_20_-HA) was also synthesized (Fig. S37–S39†). After treatment with DL-P_20_-HA, FITC-Tris was not taken up by cells at a pH range of 6.6–7.6 (Fig. S40 and S41†), which was due to the lack of helix. Based on the results stated above, DOX-NPs were expected to display different cellular-uptake behaviors outside tumor cells and at physiological pH. The cellular uptake and intracellular drug release of DOX-NPs were first evaluated by flow cytometry measurement (FCM), and free DOX·HCl was used as the control. As shown in Fig. 4c, NPs showed time-dependent internalization behavior, in which the MFI at 6 h was higher than that at 3 h. At pH 7.4, the MFI of DOX·HCl was higher than that of the other three DOX-NPs, which was because DOX·HCl was transported into cells *via* passive diffusion (Fig. 4d–f). For free DOX·HCl, the intracellular fluorescence did not change significantly upon the decrease in pH. DOX was also encapsulated by DL-P_20_-HA to form DOX-NPs, and the intracellular drug release of DOX-NPs was evaluated by FCM. Fig. S42† reveals that DOX-NPs showed lower uptake than that prepared from L-P_20_-HA, and the intracellular fluorescence did not change significantly upon the decrease in pH. However, at pH 6.6, all DOX-NPs showed enhanced MFI, which could be attributed to the improving acidity and the helical conformation promoting the cellular uptake and release of DOX (Fig. 4g–j).

**Fig. 5:**
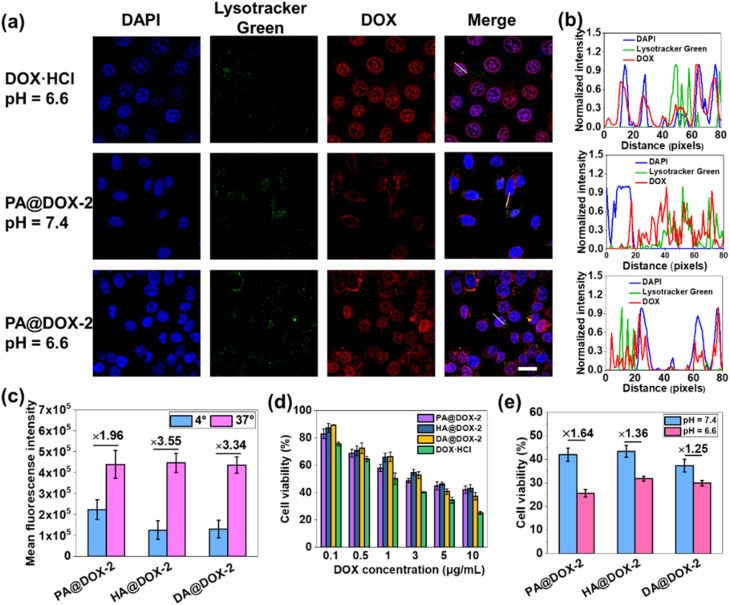
(a) CLSM images of HeLa cells treated with PA@DOX-2 or DOX·HCl at pH = 6.6 or 7.4. Scale bar = 20 μm. (b) Co-localization analysis of HeLa cells incubated with PA@DOX-2 or DOX at pH = 6.6 or 7.4, which was along the white line drawn in (a). (c) Uptake level of DOX in HeLa cells after incubation for 4 h at 4 °C or 37 °C. (d) Viability of cells treated with DOX-NPs or DOX·HCl (*n* = 3). (e) Viability of cells treated with DOX-NPs at pH 6.6 or 7.4 (*n* = 3).

Then, we studied cellular uptake and co-localization using CLSM. As shown in Fig. 5a, cells treated with DOX·HCl showed the highest nuclear red fluorescence intensity because free DOX·HCl could diffuse passively into cells. PA@DOX-2 could be internalized into cells. DOX was located mainly in LysoTracker™-labeled lysosomes, with less DOX observed in nuclei after incubation at pH 7.4. However, at pH 6.6, DOX was dominantly localized in cell nuclei, albeit with a small amount of DOX located in lysosomes (Fig. 5a). Co-localization analyses also showed that DOX diffused into nuclei more efficiently at pH 6.6 than at pH 7.4 (Fig. 5b). These results indicated that helical transition could promote internalization through non-endocytic pathways. To further evaluate the involvement of endocytosis during helix-assisted membrane penetration, we evaluated the level of cellular uptake of DOX in HeLa cells at 4 °C to block the energy-dependent endocytosis pathway. As shown in Fig. 5c and S43,† the uptake level of these DOX-NPs was inhibited markedly at low temperatures, suggesting that cellular internalization was mainly dependent on endocytosis and partial non-energy-dependent transmembrane pathways. Based on these combined results, SAi-polypeptides efficiently converted their side-chain charges from negative to neutral outside tumor cells to facilitate conformation transition from a coil to a helix, “activated” cell internalization, and promoted DOX to escape from lysosomes.

The cytotoxicity of blank NPs and DOX-NPs was evaluated using the MTT assay in HeLa cells. Obvious cytotoxicity was not observed for blank NPs, indicating that PEGylated SAi-polypeptides had excellent biosafety and biocompatibility (Fig. S44†). As expected, DOX·HCl showed the highest cytotoxicity, which was consistent with the results of cellular uptake. DOX-NPs exhibited comparable dose-dependent cytotoxicity to that of free DOX, indicating that these NPs could release DOX to kill tumor cells (Fig. 5d). To verify the feasibility of conformational transition enhancing *in vitro* cancer therapy, HeLa cells were treated with DOX-NPs in DMEM at pH 6.6 and 7.4 for cytotoxicity comparison. As shown in Fig. 5e, all DOX-NPs exhibited more efficient antitumor activity at pH 6.6, which was attributed mainly to coil-to-helix transition leading to improved uptake and DOX release.

**Fig. 6:**
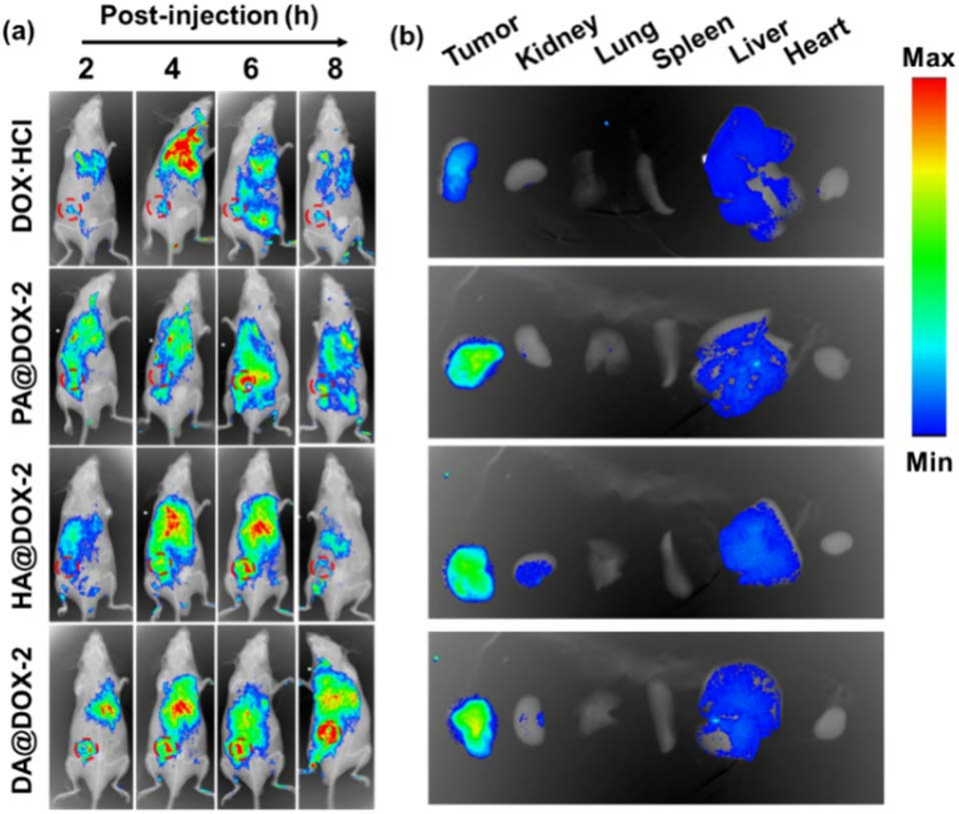
(a) *In vivo* fluorescence imaging of H22 tumor-bearing BALB/c mice after intravenous injection of NPs (6 mg kg^−1^). The tumor was marked by a circle. (b) *Ex vivo* imaging of major organs and tumors harvested at 6 h after intravenous injection.

To further explore the biodistribution of NPs, *in vivo* fluorescence imaging of tumor-bearing mice was performed after intravenous administration of DOX-NPs at different time intervals. As shown in Fig. 6a, a much stronger MFI of DOX was detected at the tumor site in DOX-NPs-treated groups. This result was mainly due to the enhanced permeability and retention (EPR) effect and helical structure-induced tumor penetration caused by tumor acidity. The major organs and tumor tissues were harvested and imaged 6 h after intravenous injection (Fig. 6b). Stronger MFI representing DOX was observed in the tumor after treatment with DOX-NPs, which further confirmed the effective accumulation of DOX-NPs in the tumor.

**Fig. 7:**
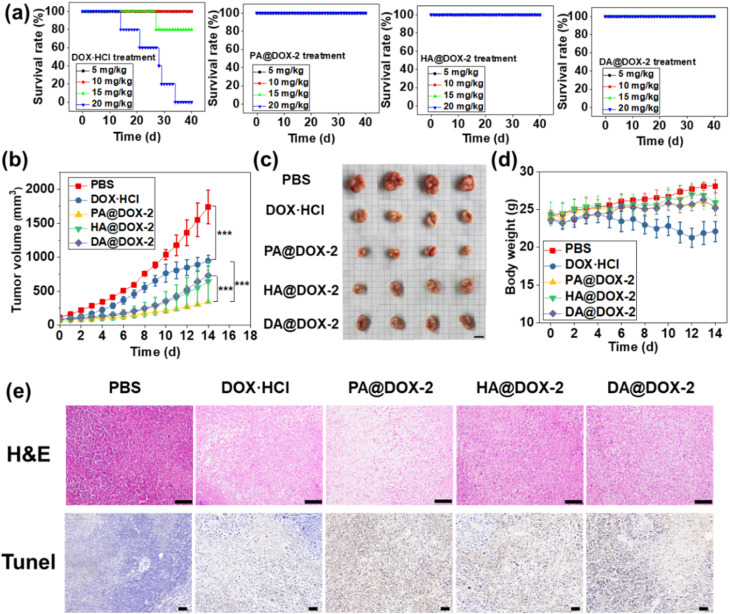
(a) MTD studies on survival for free DOX·HCl and DOX-NPs. (b) Tumor-growth curves of mice after different treatments over 14 days (*n* = 4, ***p < 0.001). (c) Images of tumors in different groups. (d) Bodyweight changes in tumor-bearing mice (*n* = 4). (e) H&E staining *via* immunohistochemistry for analyses of tissue sections. Scale bar = 1 mm. TUNEL staining: brown and blue stains indicated apoptotic and normal cells, respectively. Scale bar = 50 μm.

To investigate the maximum tolerated dose (MTD) of DOX-NPs and free DOX·HCl, a single intravenous injection was administered in tumor-free mice before the *in vivo* antitumor study (Fig. 7a). For free DOX·HCl-treated groups, mice could tolerate a dose of 10 mg kg^−1^ for up to 40 days, but increasing the dose to ∼15 mg kg^−1^ and ∼20 mg kg^−1^ resulted in 80% and 100% of mice dying, respectively. In contrast, all NPs-treated groups survived at doses up to 20 mg kg^−1^ for 40 days, indicating that DOX encapsulated in SAi-polypeptides could reduce systemic toxicity. This high MTD could be attributed to the slow release of DOX under normal physiological conditions. In addition, the slightly negative charge of the SAi domain under physiological conditions would reduce uptake by the reticuloendothelial system and enhance blood compatibility.^69^ After treatment with DOX-NPs, blood parameters and biochemistry results did not show appreciable change, further indicating the low systemic toxicity of DOX-NPs (Fig. S45 and Table S2†).

To assess the antitumor efficacy of micelles *via* intravenous injection, H22 tumor-bearing mice were divided into five groups: PBS, DOX·HCl, PA@DOX-2, HA@DOX-2, and DA@DOX-2. The dose of DOX was 5 mg kg^−1^ and DOX was injected at days 0, 3, 7, and 10. As shown in Fig. 7b, tumors in PBS-treated groups grew rapidly and the tumor size reached ∼1700 mm^3^ within 14 days. After treatment with free DOX·HCl, tumor growth was inhibited. All DOX-NPs showed higher tumor-inhibition effects than that of free DOX·HCl, likely due to the EPR effect, tumor acidity-induced penetration and drug release, prolonged circulation time, and reduced clearance. In particular, PA@DOX-2-treated groups exhibited higher inhibitory activity than the other two NPs (Fig. 7c), which was consistent with the *in vitro* results. The aromatic structure in PA@DOX-2 could also improve drug loading at physiological conditions, which could reduce unexpected drug release during circulation. Free DOX·HCl could also suppress tumor growth, but the mice experienced severe body damage, as reflected by the change in bodyweight in the DOX·HCl group. However, DOX-NPs-treated groups did not show significant body weight loss (Fig. 7d), mainly due to limited undesired DOX release in normal tissues. To further evaluate antitumor efficacy, H22 tumor-bearing mice were killed at treatment cessation, and tumor sections harvested for staining (H&E and TUNEL) (Fig. 7e). Both assays showed apoptosis and necrosis of tumor cells after treatment with free DOX·HCl and DOX-NPs. Among them, DOX-NPs showed higher activity than free DOX·HCl, which was consistent with the antitumor results. The heart, liver, spleen, lungs, and kidneys did not show obvious pathological abnormalities, further indicating the low toxicity of DOX-NPs (Fig. S46†).

## Conclusions

We disclosed the extracellular pH-responsive features of the SAi structure by systemically investigating the relationship between structure and p*K*_a_. Subsequently, a library of SAi-polypeptides was constructed *via* post-modification using sulfonyl azide and amine, which revealed pH-triggered conformation–transition behavior. Such transition pH values were in accordance with pH ranges outside the tumor, suggesting conformation transition from a random coil to a helix at the tumor site. Such random coil-to-helix transition contributed markedly to tumor penetration and cell uptake, leading to desired tumor site-specific internalization and drug accumulation. This work reveals the pH-responsive property of the SAi structure. We also designed conformation–transition polypeptides as a “smart” drug-delivery system to achieve site-specific drug accumulation. This work will enrich understanding of the relationship between versatile structures and unique biological functions, thereby providing valuable guidance for the design of biomaterials.

## Data availability

All the data supporting this article have been included in ESI.†

## Author contributions

Xiang Xu: data curation and writing (review and editing). Jinjuan Ma: writing (review and editing). Aiguo Wang: writing (review and editing). Nan Zheng: conceptualization, funding acquisition, writing (original draft), and project administration.

## Conflicts of interest

There are no conflicts of interest to declare.

## Supplementary Material

SC-015-D3SC05504C-s001
