# Supplementary material for: N-Sulfonyl amidine polypeptides: new polymeric biomaterials with conformation transition responsive to tumor acidity
Source: Chem Sci. 2023 Dec 20;15(5):1769–81. doi: 10.1039/d3sc05504c (PMC10829015; doi:10.1039/d3sc05504c)
Supplement: SC-015-D3SC05504C-s001 [file SC-015-D3SC05504C-s001.pdf]

Electronic Supplementary Material (ESI) for Chemical Science.
This journal is © The Royal Society of Chemistry 2023

N-Sulfonyl Amidine Polypeptides: A New Polymeric Biomaterial with Conformation Transition Responsive to Tumor Acidity

Xiang Xu[a,#], Jinjuan Ma[b,#], Aiguo Wang[b,*], and Nan Zheng[a,c,*]

[a] School of Chemical Engineering, Dalian University of Technology Dalian 116024, China

[b] Department of Comparative Medicine Laboratory Animal Center, Dalian Medical University Dalian 116000, China

[c] Dalian University of Technology Corporation of Changshu Research Institution, Suzhou 215500, China

* Corresponding Author

Nan Zheng: nzheng@dlut.edu.cn

Aiguo Wang: wangaiguotl@hotmail.com

[#] Authors contributed equally.

Materials and Methods

All chemicals were purchased from Energy Chemical and used as received unless otherwise specified. Methoxypolyethylene glycol amine (mPEG-NH_2, Mw = 5000 Da) was purchased from ToYong Bio Tech. Inc. (Shanghai, China). Poly-L-histidine (PLH, molecular weight ranging from 5k to 25k), Piperazine-1,4-bisethanesulfonic acid (PIPES), and 2-[4-(2-hydroxyethyl)piperazin-1-yl]ethanesulfonic acid (HEPES) were purchased from Aladdin Bio. Tech. Inc. (Shanghai, China). Triethylamine (TEA) was purchased from GuangFu Tech. Co., Ltd. (Tianjin, China). *N*, *N*-Dimethylformamide (DMF) was purified by the solvent purification system. *γ*-Propargyl-*L*-glutamate *N*-carboxyanhydride (PLG-NCA) and *γ*-propargyl-*L*-glutamate *N*-carboxyanhydride (PDLG-NCA) were synthesized according to the literatures[1]. Poly(γ-7-alkynylheptyl-*L*-glutamate) (PAHLG) was synthesized according to the literatures[2]. 3-(4,5-Dimethylthiahiazol-2-yl)-2,5-diphenyl-2H-tetrazolium bromide (MTT) was purchased from J&K Scientific. HeLa cells (human cervix adenocarcinoma cells) were grown in Dulbecco's modified Eagle medium (DMEM) containing 10% fetal bovine serum (FBS) under a 5% CO_2-containing humidified atmosphere at 37 °C. Female BALB/c mice (7-8-week-old, 25-30 g) were purchased from Dalian Medical University. The current study was ethically approved by the Institutional Animal Care and Use Committee (IACUC) of Dalian Medical University. The approval number of animal experiments is AEE22049. Procedures for animal handling and tissue samples were conducted in compliance with protocols approved by the IACUC of Dalian Medical University.

Instrument.

^{1}H NMR spectra were recorded on a Bruker AVANCE II 400 NMR spectrometer. Circular dichroism (CD) measurements were carried out on a JASCO J810 CD spectrometer. Dynamic light scattering (DLS) was performed on Malvern Zetasizer Nano ZS90 (Malvern, U.K.). Scanning electron microscopy (SEM) images were recorded on NOVA NanoSEM 450 (FEI). UV-vis absorption spectrum was measured on a Lambda 750S spectrophotometer. Fluorescence spectra were recorded on a F97pro

spectrofluorometer. Flow cytometry (FCM) was conducted on NovoCyte Flow Cytometer (NovoCyte 2060R). Intracellular imaging was captured on confocal laser scanning microscope (CLSM, FV1000, Olympus Corporation). The *in vivo* fluorescence distribution in mice bearing H22 tumors was observed at the predetermined time intervals using a KODAK In Vivo Imaging System FX PRO (Carestream Health Inc., USA) (excitation: 480 nm; emission: 535 nm). The critical micelle concentration (CMC) of the polymers was determined using pyrene as the probe. Isothermal titration calorimetry was conducted on Microcal iTC 200.

Synthesis of PPLG

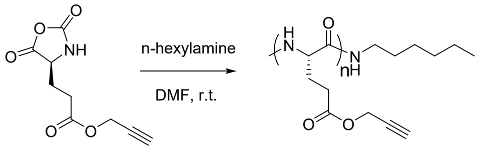

In a glovebox, PLG-NCA (170 mg, 0.805 mmol, 50 equiv.) was dissolved in 1 mL anhydrous DMF, followed by adding *n*-hexylamine stocking solution in DMF (32 μL, 0.5 M). After stirred for 2 days at room temperature, the solution was precipitated into 10-fold diethyl ether, washed with diethyl ether thrice and dried overnight under vacuum to obtain white precipitate PPLG (70% yield). The molecular weights were determined by GPC: M_w = 12221; M_n = 10465; $Đ$ = 1.16. The degree of polymerization (DP) was calculated to be 54 according to the ^{1}H NMR analysis.

Synthesis of PEG-PPLG and PEG-PPDLG

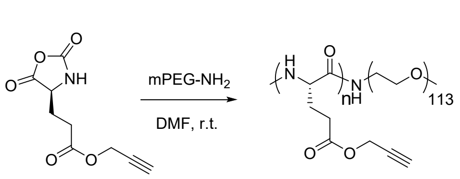

For synthesis of PEG-PPLG with target DP =25: in a glovebox, mPEG$_{5k}$-NH$_2$ (400 mg, 75.8 μmol, 1 equiv.) was dissolved in 3 mL dry DMF and PLG-NCA in 1 mL DMF (400 mg, 1.895 mmol, 25 equiv.) was added. After stirring 2 days. The solution was precipitated into 10-folds cold ether/PE (v:v = 1:1) and the precipitate was wash with ether thrice. After dried under vacuum, the white solid was obtained as PEG-PPLG.

PEG-PPDLG was prepared using PDLG-NCA as the monomer. Both the experimental DP of PEG-PPLG and PEG-PPDLG were determined as 20 according to the ^{1}H NMR analysis. It was named PEG-$PPLG_{20}$ and PEG-$PPDLG_{20}$, respectively.

PEG-$PPLG_{40}$ was synthesized using the similar method with PEG-$PPLG_{20}$ except using 40 equiv. PLG-NCA.

Synthesis of PPLG-EG_2, PPLG-EG_7, and PAHLG-EG_7

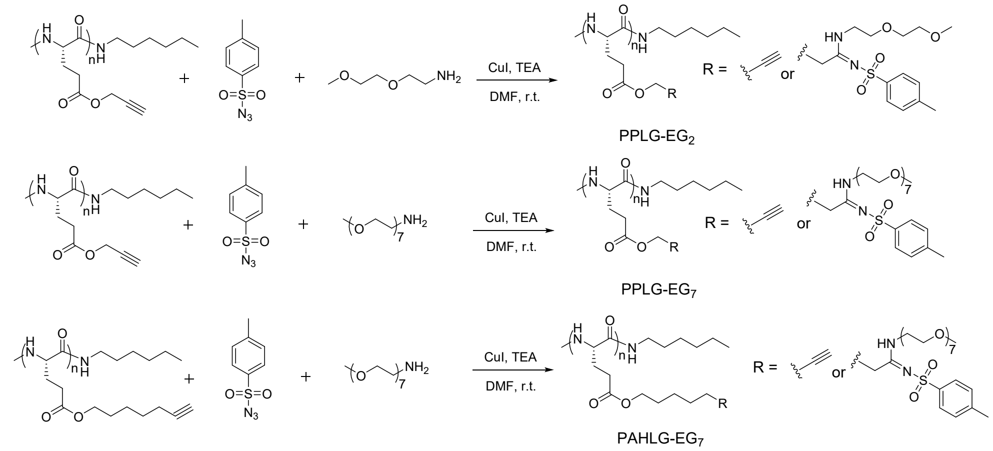

In a glovebox, PPLG (15 mg, 87.7 μmol, 1 equiv.), 2-(2-methoxyethoxy)ethanamine (32 mg, 263 μmol, 3 equiv.), CuI (3.3 mg, 17.5 μmol, 0.2 equiv.), and TEA (44 mg, 438 μmol, 5 equiv.) were dissolved in 500 μL dry DMF and *p*-toluenesulfonyl azide (47 mg, 0.24 mmol, 2 equiv.) was dropped into the solution. The solution was stirred for 12 h at room temperature, followed by precipitated into 10-fold diethyl ether thrice. After drying under vacuum, the crude product was redissolved in DMF and added EDTA-Na_2 solution to get a settled solution and dialyzed against DI water for 24 h (MWCO = 1000 Da). The DI water was changed every 6 hours. PPLG-EG_2 was obtained as white floccule after lyophilization (50% yield). The SAi modified rate was calculated as 60% according to the ^{1}H NMR analysis. PPLG-EG_7 was synthesized with the similar method to PPLG-EG_2 except for using PEG_{350}-NH_2 (48% yield). The SAi modified rate was about 53% according to ^{1}H NMR analysis. PAHLG-EG_7 was synthesized with the similar method with PPLG-EG_7 except for using PAHLG. The SAi modified rate was about 100% according to ^{1}H NMR analysis.

Synthesis of PPLG-Bu

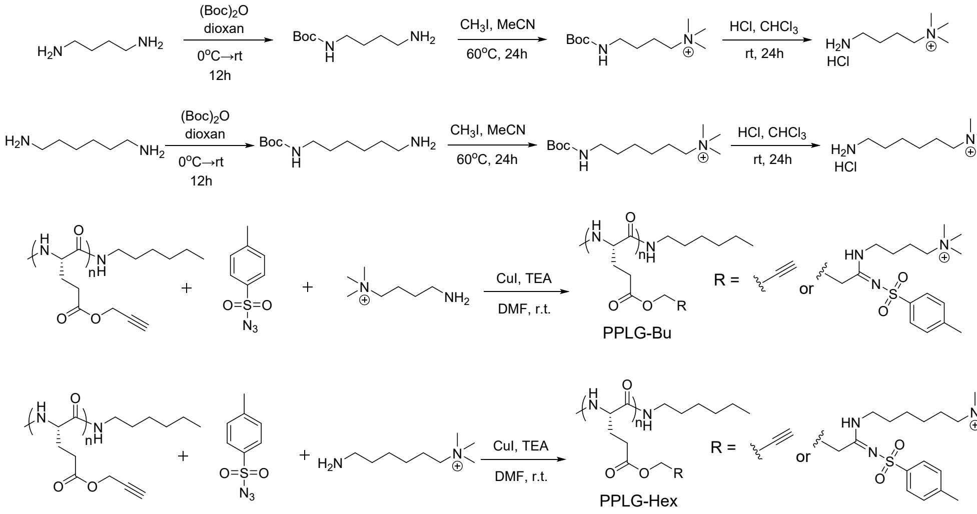

After 1,4-butanediamine (2 mL, 20 mmol, 3.5 equiv.) was dissolved in 15 mL 1,4-dioxane, di-tert-butyl decarbonate (1.2 g, 5.6 mmol, 1 equiv.) in 15 mL 1,4-dioxane was added drop by drop at 0 °C and the mixture was stirred for 16 h at room temperature. After filtration, the solvent and residual 1,4-butanediamine were removed through rotary evaporation. Excessive H_2O was added and the precipitate was removed. The filtrate was extracted using DCM thrice and the combined fraction was washed once with saturated NaCl solution. After treated by anhydrous Na_2SO_4, the supernatant was collected. The product *N*-(*tert*-butoxycarbonyl)-1,4-butanediamine was obtained after the solvent was removed (80% yield).

N-(*tert*-Butoxycarbonyl)-1,4-butanediamine (700 mg, 3.72 mmol, 1 equiv.) and K_2CO_3 (1.962 g, 14.2 mmol, 3.8 equiv.) were stirred in 20 mL MeCN at room temperature for 30 min. CH_3I (1.845 g, 13 mmol, 3.5 equiv.) was then added and the mixture was heated to 85 °C, followed by reaction for 20 h. After cooling to room temperature, the K_2CO_3 was removed by filtration. The filtrate was concentrated and excessive diethyl ether was added for stirring and sonication to obtain white precipitate 4-[[(1,1-dimethylethoxy)carbonyl]amino]-*N*,*N*,*N*-trimethyl-1-butanaminium (88% yield).

4-[[(1,1-Dimethylethoxy)carbonyl]amino]-*N*,*N*,*N*-trimethyl-1-butanaminium (600 mg, 2.6 mmol) was dissolved in 13 mL $CHCl_3$, followed by adding 260 μL concentrated HCl solution. The mixture was stirred overnight and the solvent was then

removed to get brown solid. Such solid was redissolved in H_2O, and neutralized by Na_2CO_3 solution. After the removal of the precipitate, the filtrate was dried and redissolved in methanol. The supernatant was collected and concentrated to obtain white solid 4-amino-*N*, *N*,*N*-trimethyl-1-butanaminium (60% yield).

In a glovebox, PPLG (20 mg, 117 μmol, 1 equiv.), 4-amino-*N*,*N*,*N*-trimethyl-1-butanaminium (78 mg, 469 μmol, 4 equiv.), CuI (4.4 mg, 23.4 μmol, 0.2 equiv.), and TEA (60 mg, 585 μmol, 5 equiv.) were dissolved into 600 μL dry DMF. Then *p*-toluenesulfonyl azide (45 mg, 234 μmol, 2 equiv.) was dropped into the solution. After stirred for 12 h, the solution was precipitated into 10-fold diethyl ether thrice and dried under vacuum. The crude product was redissolved in DMF and added EDTA-Na_2 solution to get settled solution and dialyzed against DI water for 24 h (MWCO = 1 kDa). The DI water was changed every 6 hours. PPLG-Bu was obtained as white solid (56% yield). SAi modified rate was about 47% according to ^{1}H NMR analysis.

PPLG-Hex was synthesized starting from 1,6-hexanediamine through similar routes with PPLG-Bu. SAi modified rate was about 58.8% according to ^{1}H NMR analysis.

Synthesis of PEG-PPLG bearing SAi structure

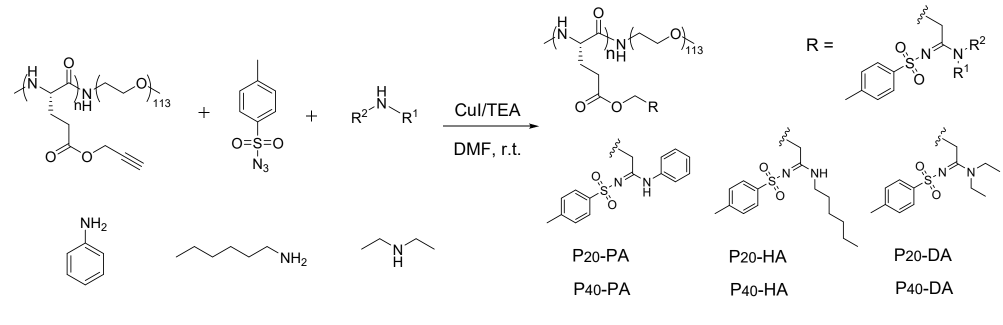

PEG-PPLG$_{20}$ (100 mg, numbers of alkyne = 0.257 mmol, 1 equiv.), phenylamine (72 mg, 0.77 mmol, 3 equiv.), CuI (9.8 mg, 51.4 μmol, 0.2 equiv.), and TEA (130 mg, 1.285 mmol, 5 equiv.) were dissolved into 2.5 mL dry DMF. Then *p*-toluenesulfonyl azide (102 mg, 515 μmol, 2 equiv.) was dropped into the solution. After stirring for 12 h, the solution was precipitated into 10-fold diethyl ether thrice and dried under vacuum. The crude product was redissolved in DMF and added EDTA-Na_2 solution,

followed by dialyzed against DI water for 24 h (MWCO = 1 kDa). The DI water was changed every 6 hours. P_{20}-PA was obtained as a white solid. Under similar conditions, P_{20}-HA was prepared using *n*-hexylamine and P_{20}-DA was prepared using diethylamine. As a control, DL-P_{20}-HA was prepared using a similar method to P_{20}-HA using PEG-$PPDLG_{20}$. P_{40}-(PA, HA, DA) were prepared with a similar method. The SAi-modified rates were shown in **Table S1.**

Table S1. Characterization of polymers.

Entry	Samples	DP[a]	SAi Modified rate (%)[a]
1	PPLG	54	/
2	PEG-$PPLG_{20}$	20	/
3	PEG-$PPLG_{40}$	40	/
4	P_{20}-PA	20	51.6
5	P_{20}-HA	20	50.6
6	P_{20}-DA	20	47.5
7	P_{40}-PA	40	57.4
8	P_{40}-HA	40	57.6
9	P_{40}-DA	40	47.5
10	DL-P_{20}-HA	20	49.3

[a] Determined by ^{1}H NMR.

Synthesis of SAi small molecules

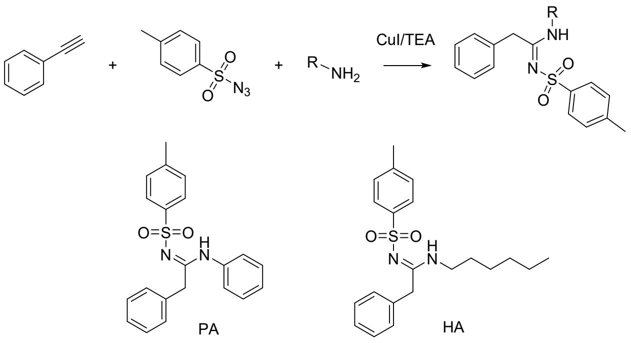

SAi small molecules (PA and HA) were synthesized according to previous reports[3]. The yield was 67% and 64.8% for PA and HA, respectively.

PA, ^{1}H NMR ($CDCl_3$): δ 7.85-7.91 (q, 2H), 9.98 and 7.46-6.76 (m,15H), 4.51,3.79, and 3.61 (s, 2H)

HA, ^{1}H NMR ($CDCl_3$): δ 7.9-7.82 (m, 2H), 7.4-7.17 (m, 2H), 5.21 (s, 2H), 4.3 and 3.67 (s, 2H), 3.26-3.17 (m, 2H), 2.44 (s, 3H), 1.44-1.14 (m, 8H), 0.84 (m, 2H);

The protonation degree at different pH conditions

The polypeptides (6 mg) were dissolved in HCl solution (6 mL) with initial pH about 2.6 and titrated by adding small volumes (5 or 10 μL increment) of NaOH (0.1 M). The pH values were measured with a pH meter in real time. The pH value was correlated with the volume of NaOH solution and the corresponding first derivative was obtained. The extreme point of the first derivative was the starting point and end point of protonation, so as to calculate the protonation degree of the polypeptides.

CD analysis at different pH conditions

The pH value was adjusted by adding a specific volume of concentrated HCl or NaOH and measured with a pH meter. After adjusting to the desired pH, the polypeptides aqueous solution (0.025 mg/mL) was placed in a quartz cell with a path length of 1.0 cm and measured at room temperature. The molar ellipticities were calculated in deg $\times cm^2 \times dmol^{-1}$. The helicity was calculated using the equation: $[\theta]_\lambda = (MRW \times \theta_\lambda)/(d \times c)$, helicity= $(-[\theta]_{222}+3000)/39000$. MRW is the mean residue weight, θ_λ is the observed ellipticity at wavelength λ (i.e. 222 nm), d is the path length (mm) and c is the concentration (mg/mL).

Drug loading of polypeptide micelles.

The drug-loaded nanoparticles (NPs) were prepared by the nanoprecipitation method. DOX·HCl was first neutralized by TEA (1.5 molar equivalents to DOX) in DMF. P_{20}-PA, P_{20}-HA, or P_{20}-DA were dissolved in DMF and added to the DOX solution. The mixture was stirred for 2 h in the dark, and then PBS (pH= 7.4, 0.1 M) was added dropwise into the mixture under vigorous stirring. The mixture was stirred in the dark overnight. DMF and unencapsulated were removed by dialysis against PBS for 6 h and DI water for 18 h (MWCO = 3000 Da). The NPs were obtained after lyophilization. The absorbance at 480 nm by the UV-Vis spectrometer was used to

determine the DOX content. The drug loading content (DLC, wt%) and drug loading efficiency (DLE, wt%) were calculated according to the following formula:

DLC = (amount of loaded drug/amount of drug-loaded NPs) × 100%

DLE = (amount of loaded drug/amount of feeding drug) × 100%

Characterization of size and zeta potential

The SAi polypeptides and drug-loaded NPs were dissolved in DI water with a concentration of 1 mg/mL for the analysis of DLS. The HCl solution (0.1 M) and NaOH (0.1 M) were used to adjust the pH. Then the size and distribution were analyzed by DLS directly without further purification.

The pH titration and protonation degree of SAi polypeptides at different pH conditions

SAi polypeptides were dissolved in HCl solution (1 mg/mL, 6 mL) and titrated by adding small volumes (5 μL increment) of NaOH solution (0.1 M). The pH values of the solution were measured by pH meter in real-time. The pH value was correlated with the volume of NaOH solution and the corresponding first derivative was obtained. The extreme point of the first derivative was the starting point and end point of protonation, to calculate the protonation degree of the SAi polypeptides.

In vitro DOX release

The release of DOX from NPs was studied using the dialysis method in PBS solution containing 0.5% tween 80 (pH = 5.5, 6.8, 7.4). Briefly, DOX-NPs were dispersed in a dialysis bag (5 mL, containing 1 mg DOX), and then immersed in 65 mL release medium, which was performed at 37 °C under stirring (200 rpm). At pre-determined time intervals, a 5 mL release medium was withdrawn and another fresh release medium was added. The DOX amount was determined by spectrofluorimetry (excitation: 480 nm; emission: 590 nm).

Membrane activity.

Cells were seeded on 96-well plates at 1×10^4 cells/well and cultured for 16 h. The medium was replaced by a serum-free medium, into which NPs and FITC-Tris were added at 2 μg/well and 1 μg/well, respectively. Free FITC-Tris without treatment by NPs was a control. After incubation at 37 °C under different pH for 2 h, cells were washed with PBS solution for 3 times and then lysed with RIPA lysis buffer (100 μL/well). The number of FITC-Tris in the cell lysate was quantified by spectrofluorimetry (excitation: 488 nm; emission: 525 nm). The FITC uptake level was represented by the fluorescence intensity. Cells incubated with free FITC-Tris in the absence of polypeptides were included as a negative control. Hela cells were seeded on a glass bottom petri dish at 1×10^5 cells/well (1 mL) and cultured for 16 h, into which NPs and FITC-Tris were added at 20 μg/well and 10 μg/well. After washed with PBS three times, the cells were subjected to observation using CLSM.

In vitro cellular uptake

Hela cells were seeded on a glass bottom petri dish at 1×10^5 cells/well (1 mL) and cultured for 16 h. After incubating with NPs (100 μL, 100 μM) for 4 h, the cells were washed with PBS three times. Then the cells were firstly stained with Lysotracker Green (60 nM), then fixed with paraformaldehyde (4%), stained with DAPI (2 μg/mL), and subjected to observation using CLSM. Co-localization analysis was conducted using the software Image J after selecting the region. To simulate the tumor pH microenvironment, the cell culture medium was adjusted to pH 7.4 and 6.6 using PIPES (25 mM) and HEPES (25 mM), respectively[4, 5].

FCM analysis

The cells were seeded on 6-well plates at 2×10^6 cells/well and treated with DOX·HCl and DOX-NPs at pH 6.6 and 7.4. After incubation for 3 or 6 h, the culture medium was removed and the cells were washed with PBS. The cells were digested with trypsin and washed three times with PBS. The cells were resuspended in 750 μL PBS after centrifugation (1000 rpm/min, 5 min) and filtered through a 40 μm nylon

mesh to remove cell aggregates before measurements were made. The mean fluorescence intensity of DOX in cells was analyzed by FCM (excitation: 488 nm; emission: 585 nm).

In vitro antitumor efficacy

Hela cells were seeded on 96-well plates at 1×10^4 cells/well and cultured for 16 h. 10 μL of free DOX·HCl, DOX-NPs, and NPs solution at different concentrations were added and incubated for 48 h. The cells were subjected to an MTT assay to determine the cytotoxicity.

In vivo fluorescence imaging and biodistribution.

Female BALB/c mice (7-8 weeks old, 20-25 g) were bred and all experiments were performed in the Laboratory Animal Center of Dalian Medical University. H22-bearing mice were obtained by subcutaneous injection of H22 cells collected from the peritoneal cavity of the BALB/c mice after 6 d. Ascites (100 μL) containing H22 cells (1×10^7 cells in total) were subcutaneously injected into the BALB/c mice at the back near the right flank. Mice were intravenously injected with DOX-NPs after the tumor volume of mice reached around 50 mm^3 (2.5 mg DOX/kg). The *in vivo* fluorescence distribution in mice bearing H22 tumors was observed at the predetermined time intervals (2, 4, 6, and 8 h) using a KODAK In Vivo Imaging System FX PRO (Carestream Health Inc., USA) (excitation: 488 nm; emission: 535 nm). In another parallel study, mice were sacrificed at 6 h post-injection, and the major organs (heart, liver, spleen, lung, and kidney) as well as tumors were collected and imaged.

Maximum tolerated dose (MTD) study

BALB/c male mice were divided into 16 groups (n = 5) and intravenously injected once with free DOX·HCl or DOX-NPs (the doses of DOX were 5, 10, 15, 20 mg/kg). Changes in body weight and survival of mice were followed for 40 days. The MTD was defined as the dose that causes neither mouse death due to toxicity nor greater than 15%

body weight loss or other remarkable changes in the general appearance within the entire period of the experiments.

Biocompatibility analysis

The biocompatibility of DOX-NPs was evaluated in terms of serum biochemical analyses. These were intravenously injected into BALB/c mice at DOX doses of 10 mg/kg, and blood was collected at 24 h post-injection. The serum levels of total protein (TP), aspartate aminotransferase (AST), alanine aminotransferase (ALT), alkaline phosphatase (ALP), blood urea nitrogen (BUN), and creatinine (CR) were measured. The hematology test evaluated the following parameters: white blood cells (WBC), neutrophils (Neu), lymphocytes (Lym), monocytes (Mon), eosinophils (Eos), basophils (Bas), red blood cells (RBC), hemoglobin (HGB), hematocrit (HCT), mean corpuscular volume (MCV), mean corpuscular hemoglobin (MCH), mean corpuscular hemoglobin concentration (MCHC), platelets (PLT), mean platelet volume (MPV), platelet distribution width (PDW), and platelet crit (PCT).

In vivo antitumor efficacy

Female BALB/c mice (7-8 weeks old, 20-25 g) were bred and all experiments were performed in the Laboratory Animal Center of Dalian Medical University. H22-bearing mice were obtained by subcutaneous injection of H22 cells collected from the peritoneal cavity of the BALB/c mice after 6 d. Ascites (100 μL) containing H22 cells (1×10^7 cells in total) were subcutaneously injected into the BALB/c mice at the back near the right flank. After the tumor volume of mice reached around 100 mm^3, mice were divided into 5 groups (PBS, DOX·HCl, PA@DOX-2, HA@DOX-2, and DA@DOX-2) for intravenous injection (The doses of the DOX moieties were 5 mg/kg per mouse). Mice were intravenously injected on the 0, 3rd, 7th and 10th day. The body weight and tumor sizes were recorded every day. The tumor volume was calculated as $V = 0.5 \times (\text{tumor length}) \times (\text{tumor width})^2$. The experiment finished on the 15th day and mice were sacrificed. Major organs including the heart, liver, spleen, lung, kidney,

and tumor were harvested. The hematoxylin and Eosin (H&E) method was used for histological evaluation and transferase-mediated UTP end labeling (Tunel) assay was used to evaluate tumor cell apoptosis.

Characterization

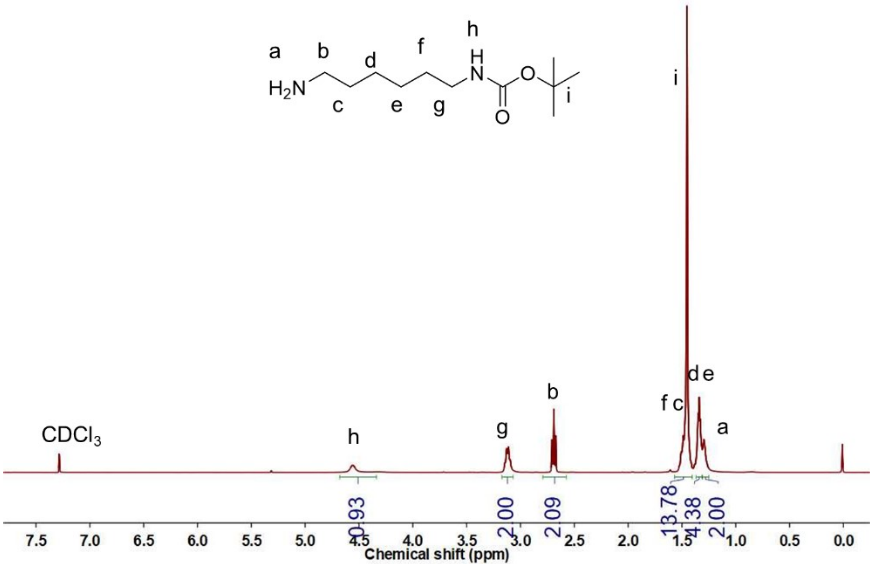

Figure S1. ^{1}H NMR spectrum of *tert*-butyl *N*-(6-aminohexyl) carbamate in $CDCl_3$.

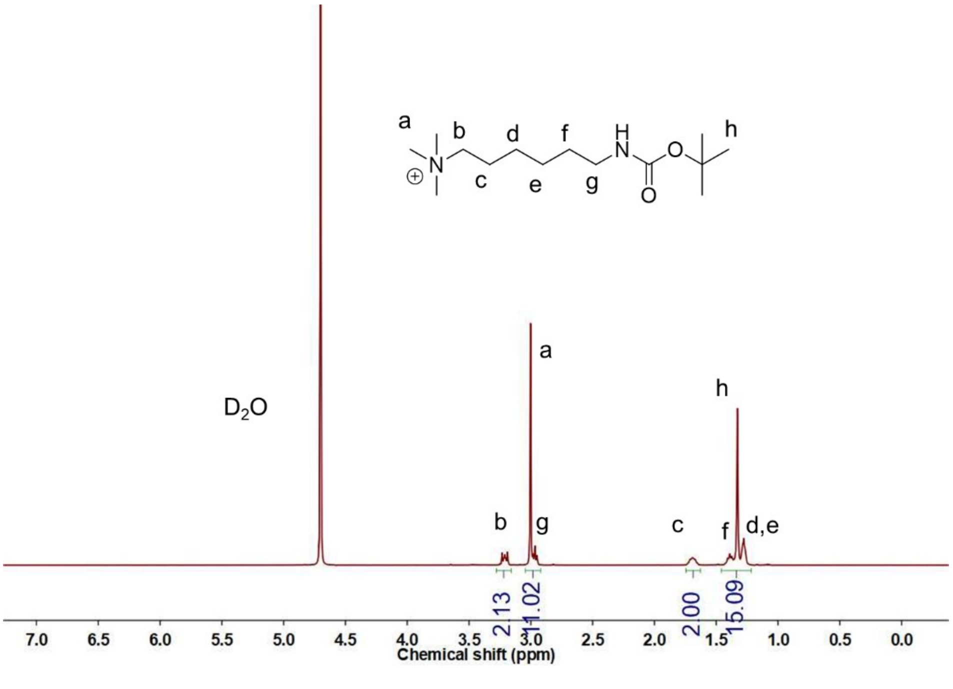

Figure S2. ^{1}H NMR spectrum of 6-((tert-butoxycarbonyl)amino)-*N*,*N*,*N*-trimethylhexan-1-aminium in D_2O.

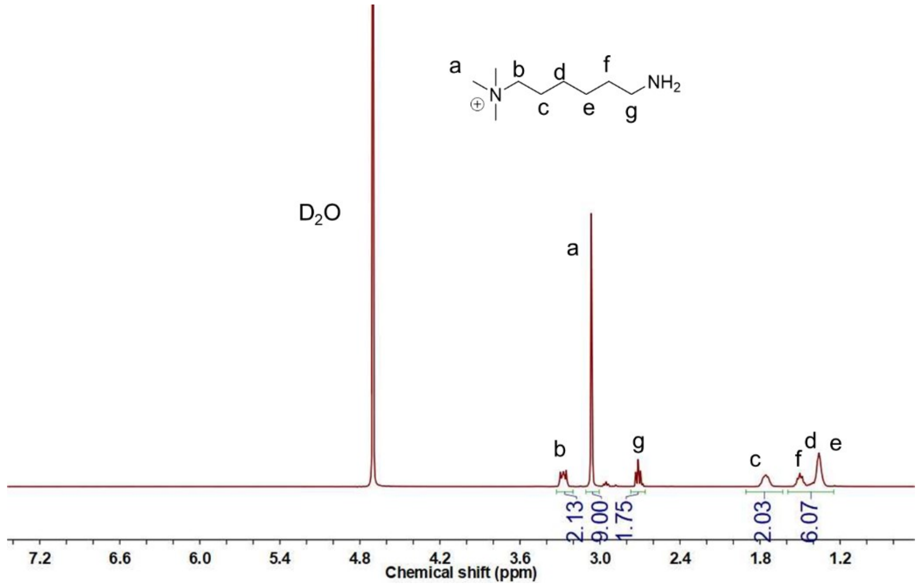

Figure S3. ^{1}H NMR spectrum of 6-amino-*N*,*N*,*N*-trimethylhexan-1-aminium in D_2O.

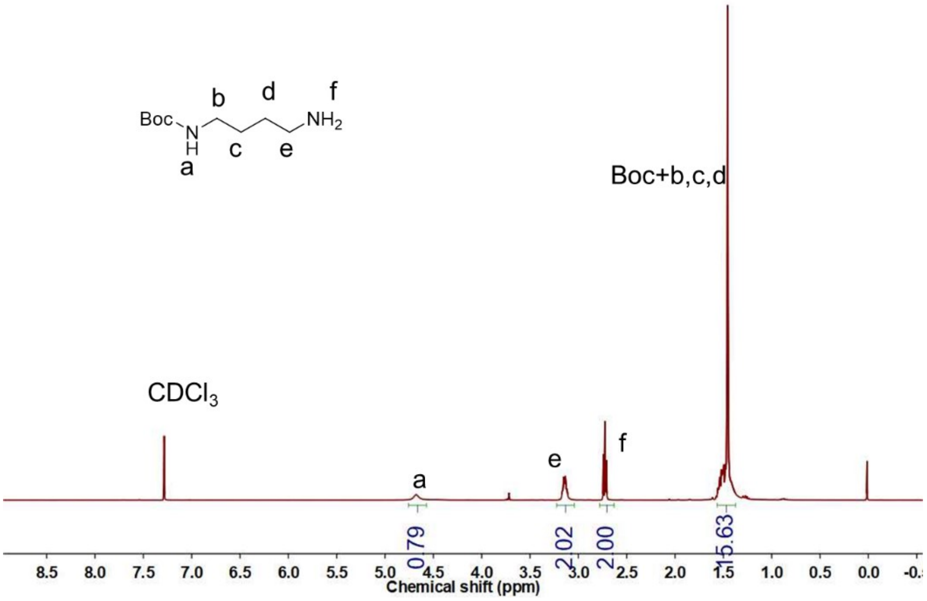

Figure S4. ^{1}H NMR spectrum of *N*-(*tert*-butoxycarbonyl)-1,4-butanediamine in $CDCl_3$.

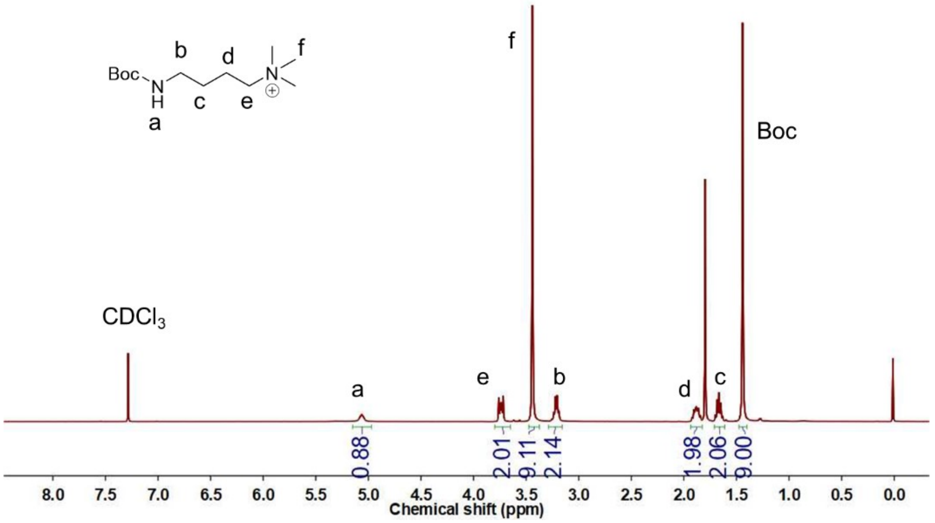

Figure S5. ^{1}H NMR spectrum of 4-[[(1,1-dimethylethoxy)carbonyl]amino]-*N*,*N*,*N*-trimethyl-1-butanaminium in $CDCl_3$.

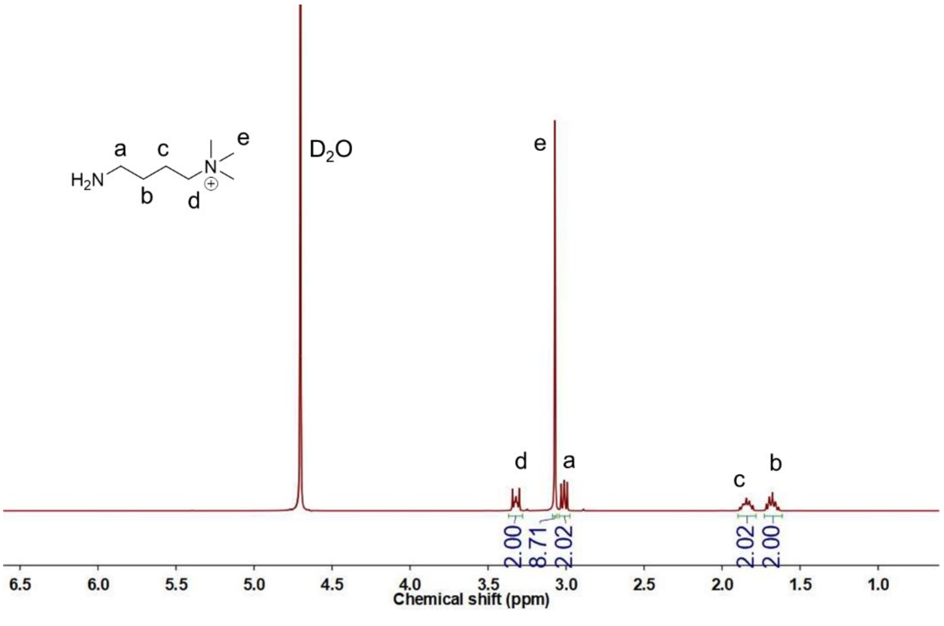

Figure S6. ^{1}H NMR spectrum of 4-amino-*N*,*N*,*N*-trimethyl-1-butanaminium in D_2O.

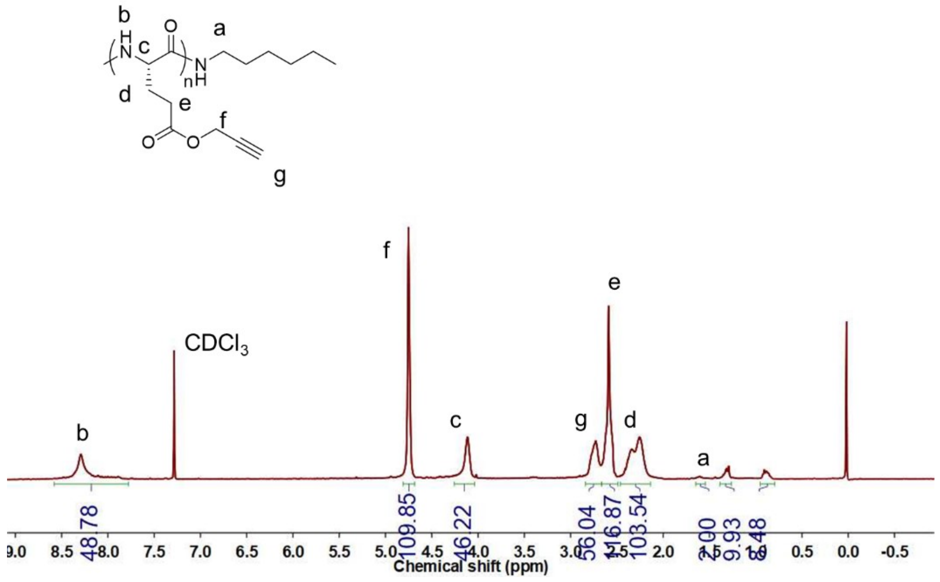

Figure S7. ^{1}H NMR spectrum of PPLG in $CDCl_3$.

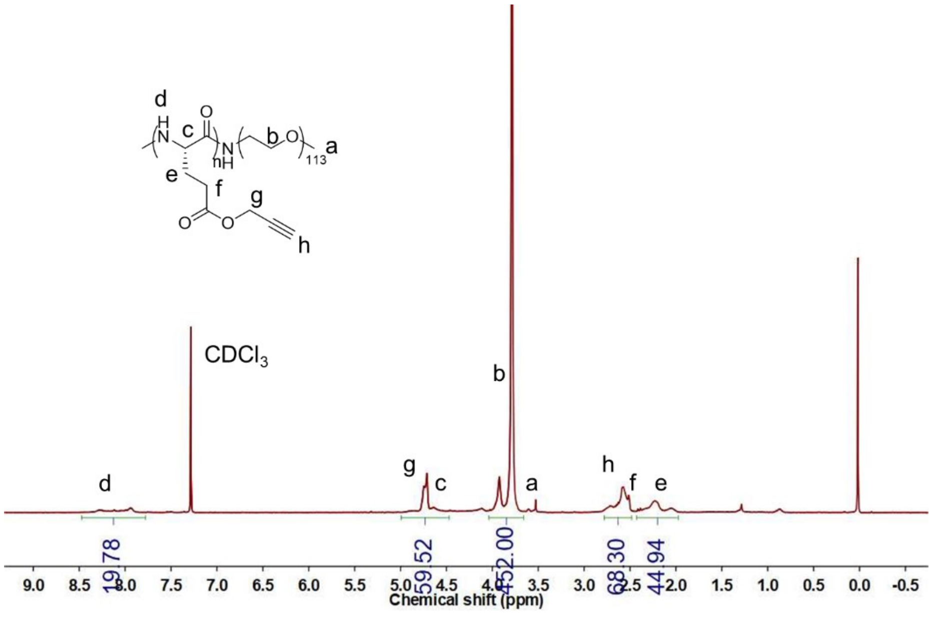

Figure S8. ^{1}H NMR spectrum of PEG-$PPLG_{20}$ in $CDCl_3$.

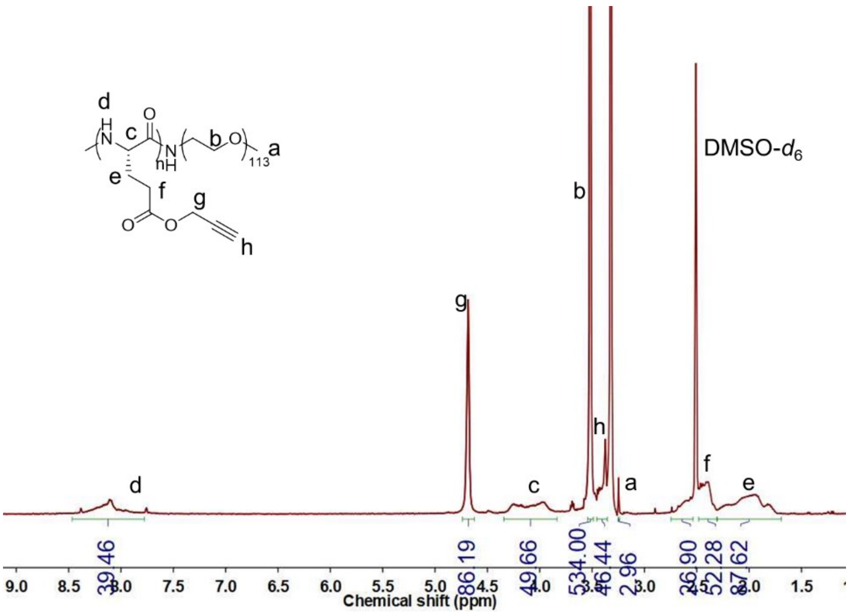

Figure S9. ^{1}H NMR spectrum of PEG-PPLG$_{40}$ in DMSO-d_6.

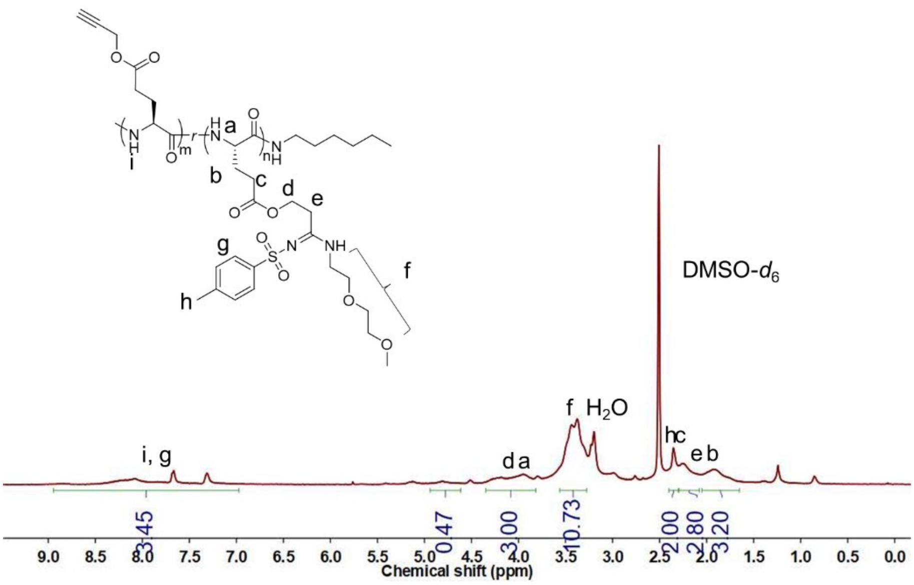

Figure S10. ^{1}H NMR spectrum of PPLG-EG$_2$ in DMSO-d_6.

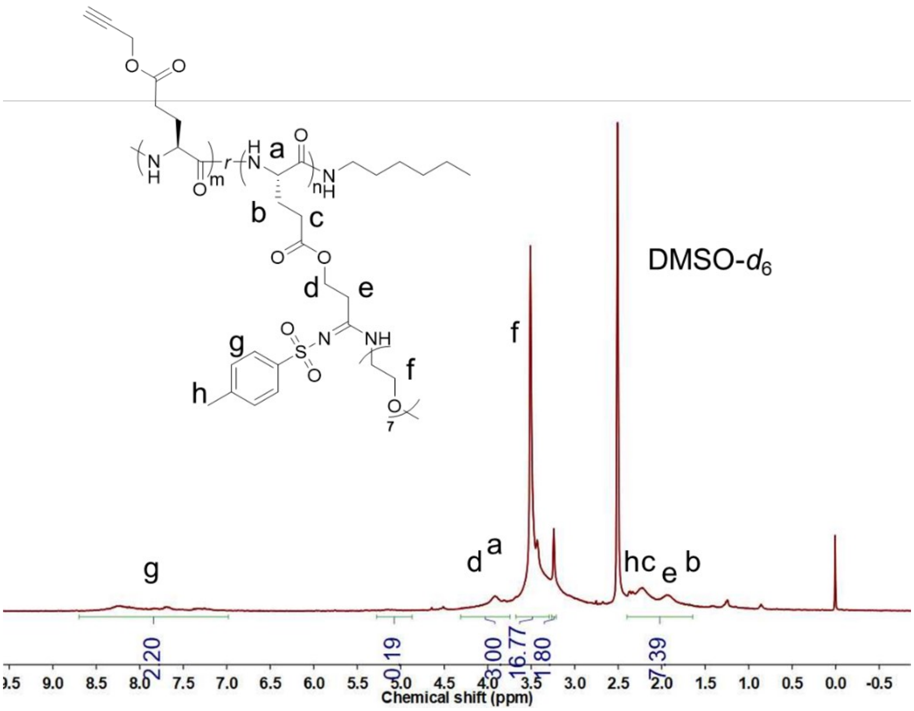

Figure S11. ^{1}H NMR spectrum of PPLG-EG$_7$ in DMSO-d_6.

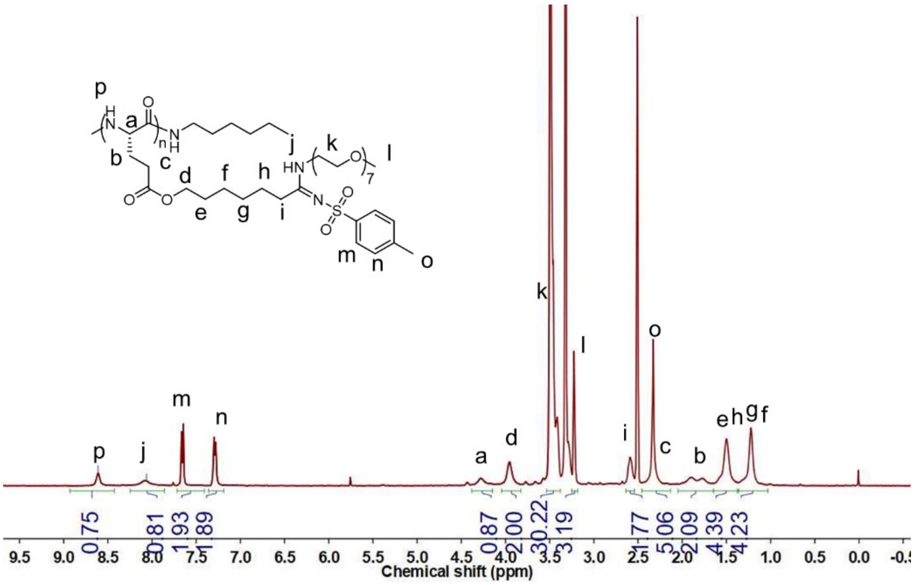

Figure S12. ^{1}H NMR spectrum of PAHLG-EG$_7$ in DMSO-d_6.

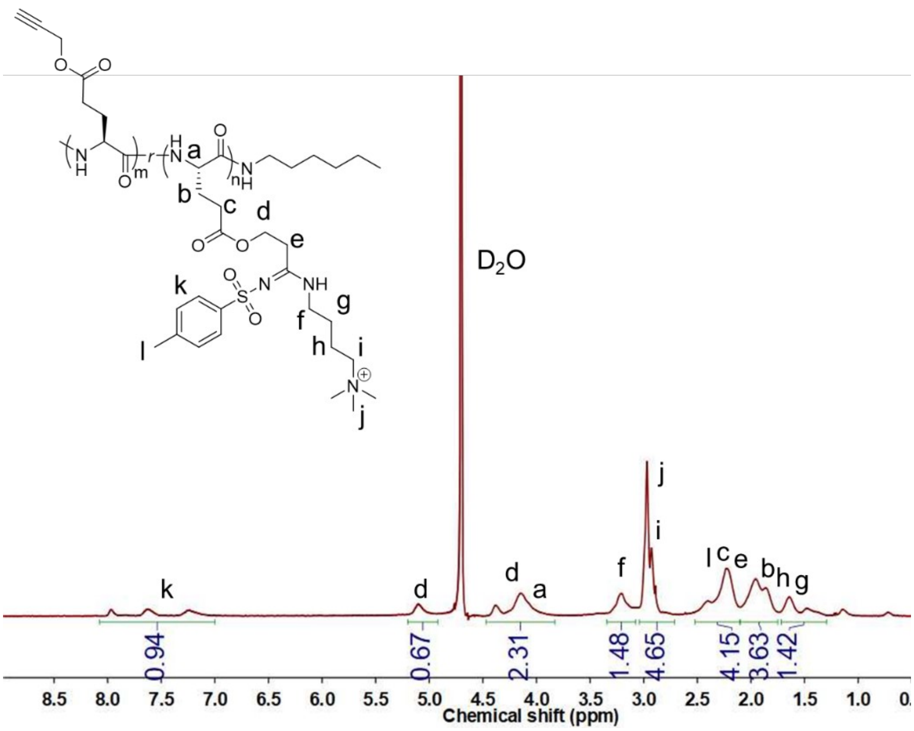

Figure S13. ^{1}H NMR spectrum of PPLG-Bu in D_2O. The integral of peak k was not enough, which may be the amphiphilicity causing the benzene ring in the hydrophobic core[6].

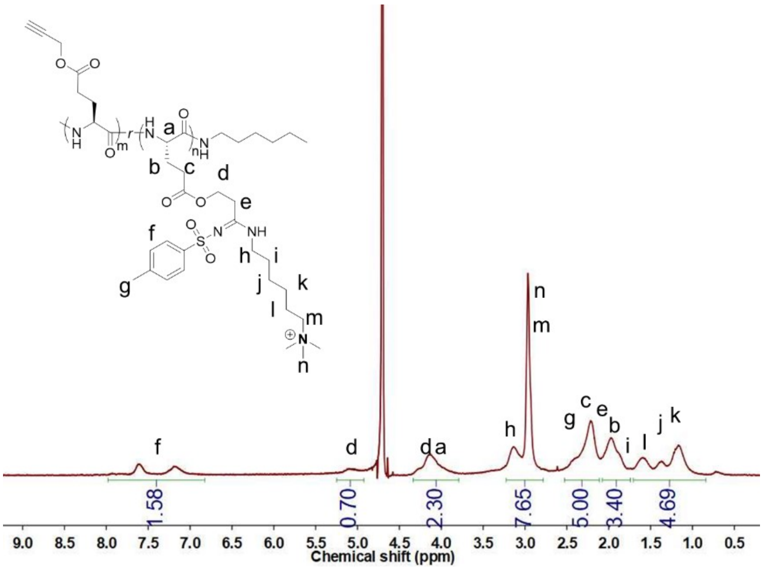

Figure S14. ^{1}H NMR spectrum of PPLG-Hex in D_2O. The integral of peak f was not enough, which may be the amphiphilicity causing the benzene ring in the hydrophobic core.

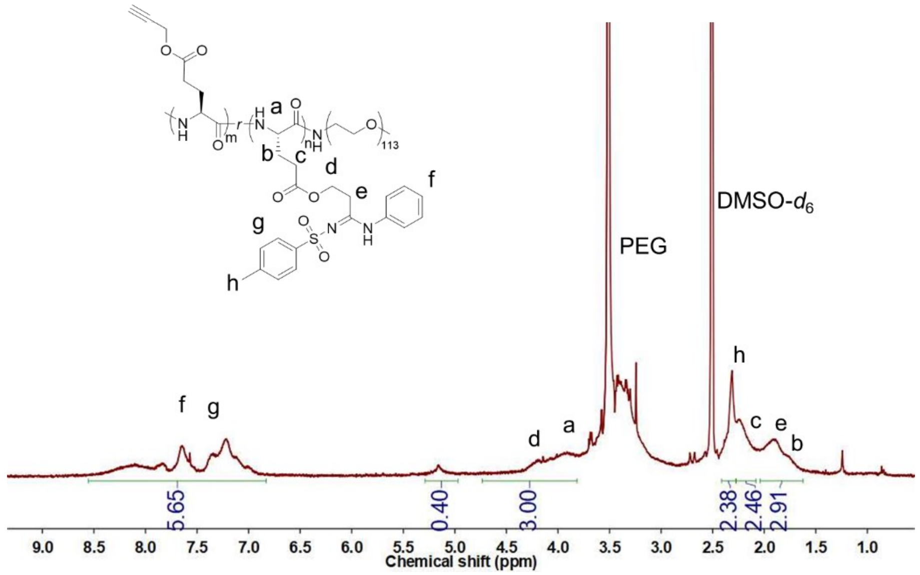

Figure S15. ^{1}H NMR spectrum of P_{20}-PA in DMSO-d_6.

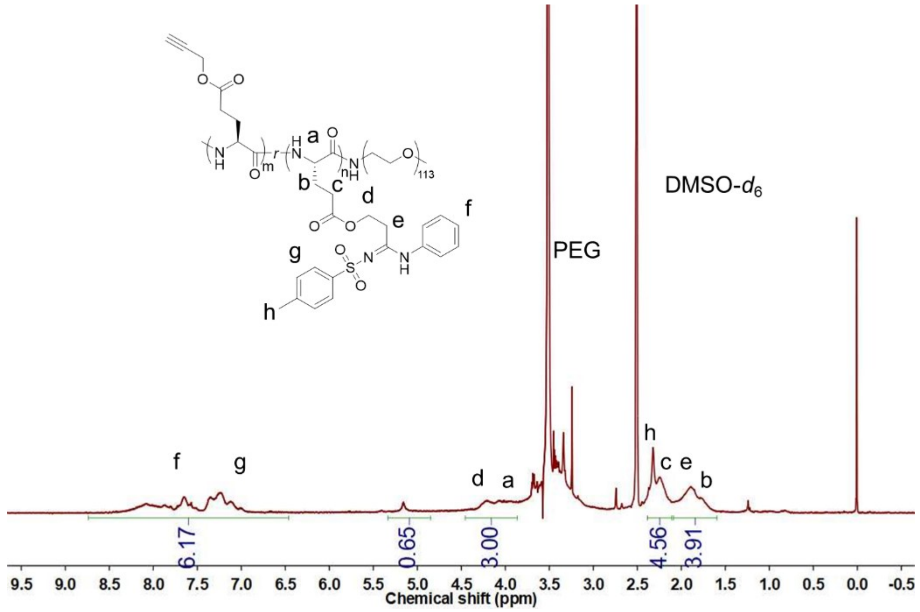

Figure S16. ^{1}H NMR spectrum of P_{40}-PA in DMSO-d_6.

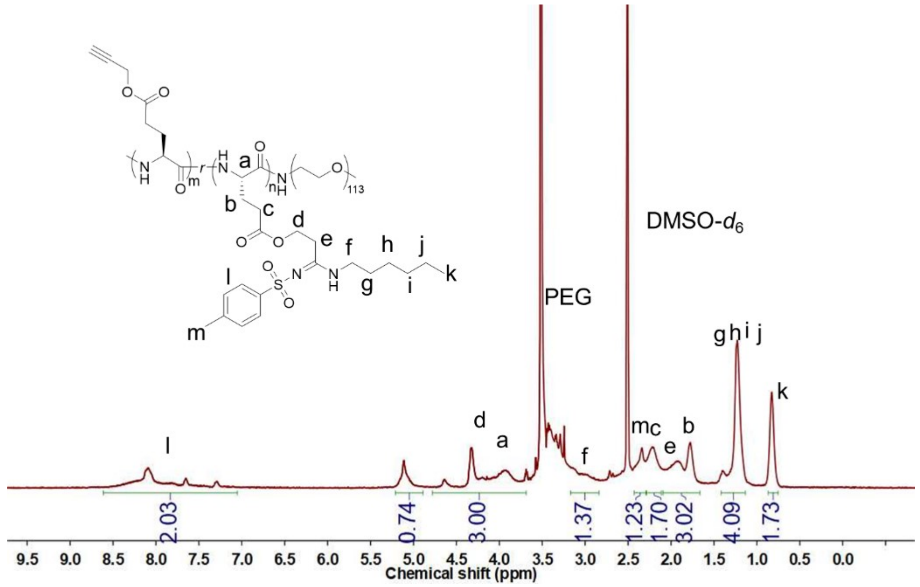

Figure S17. ^{1}H NMR spectrum of P_{20}-HA in DMSO-d_6.

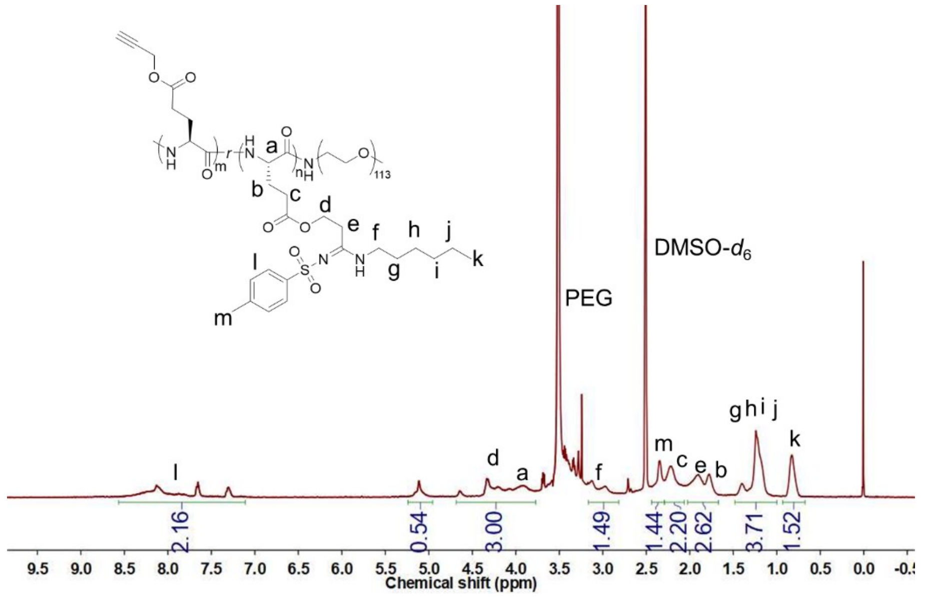

Figure S18. ^{1}H NMR spectrum of P_{40}-HA in DMSO-d_6.

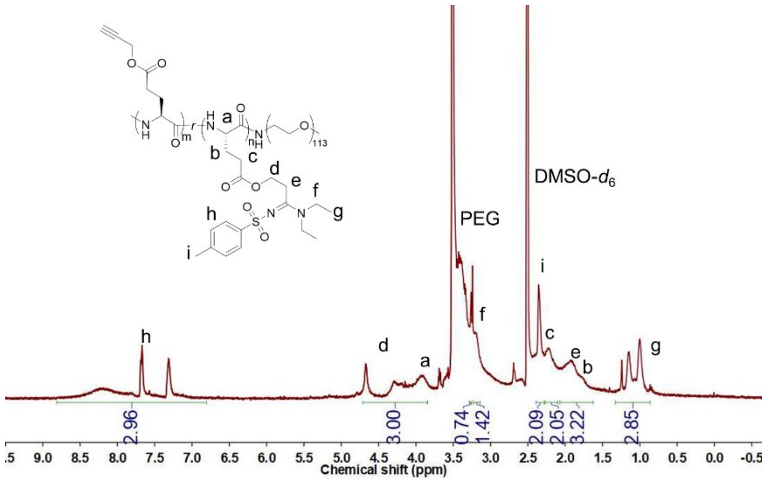

Figure S19. ^{1}H NMR spectrum of P_{20}-DA in DMSO-d_6.

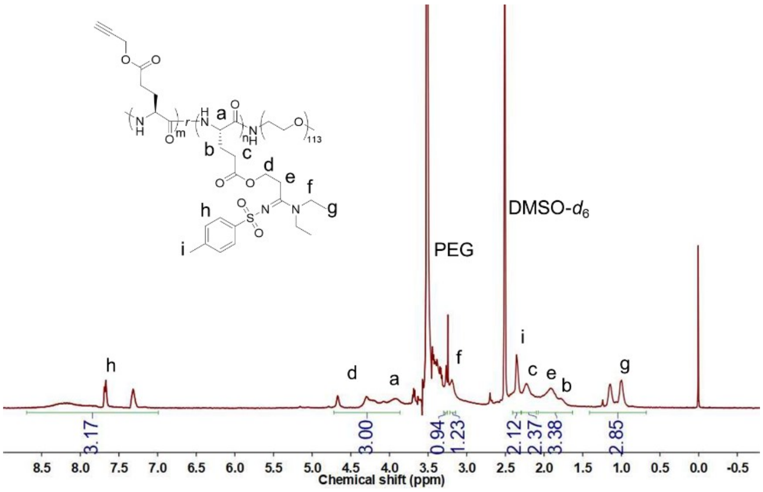

Figure S20. ^{1}H NMR spectrum of P_{40}-DA in DMSO-d_6.

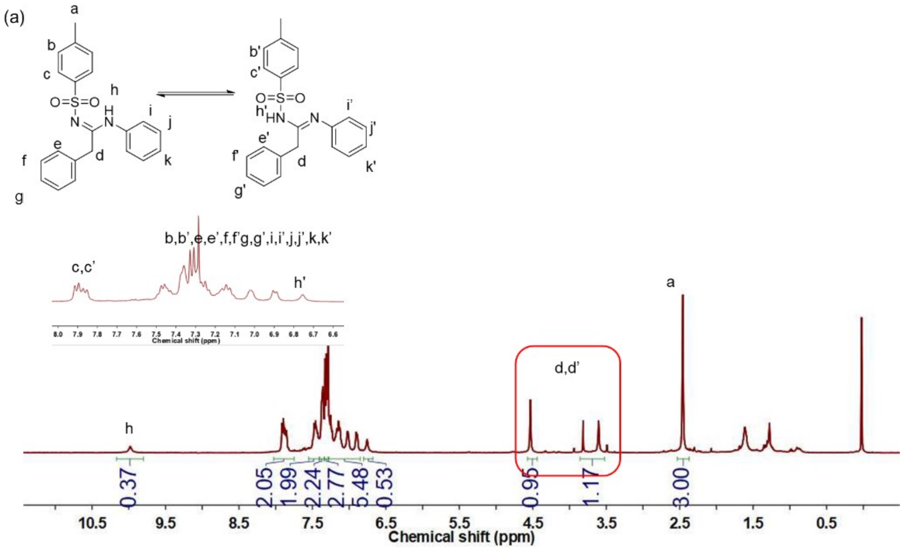

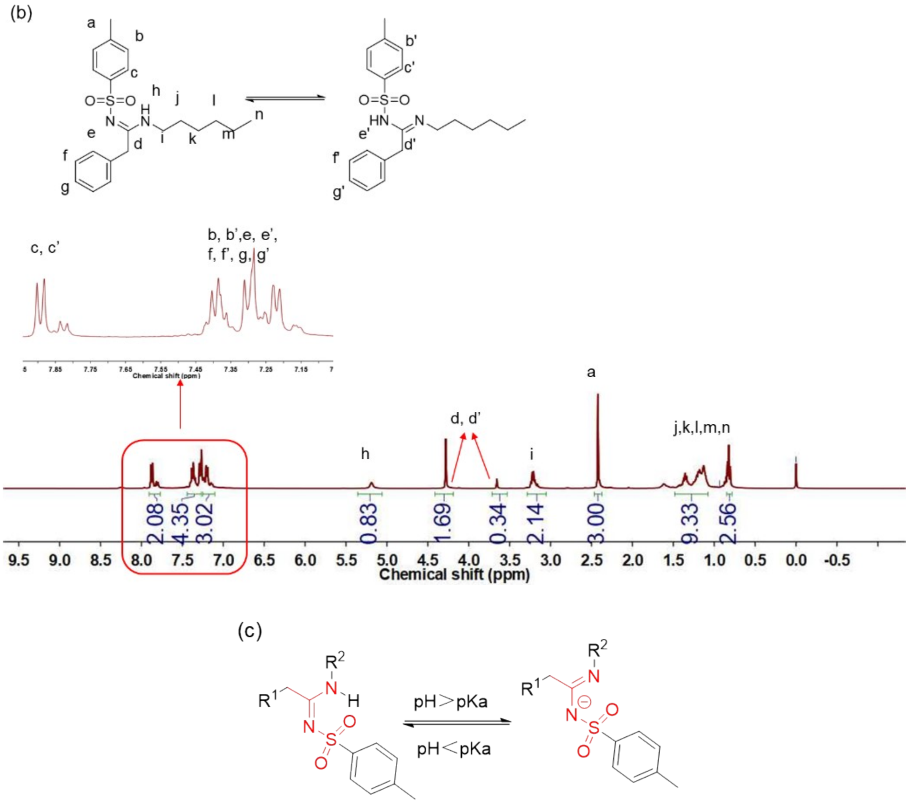

Figure S21. ^{1}H NMR spectra of small molecules of SAi in $CDCl_3$: (a) PA, (b) HA. (c) Resonance structure of ionized SAi.

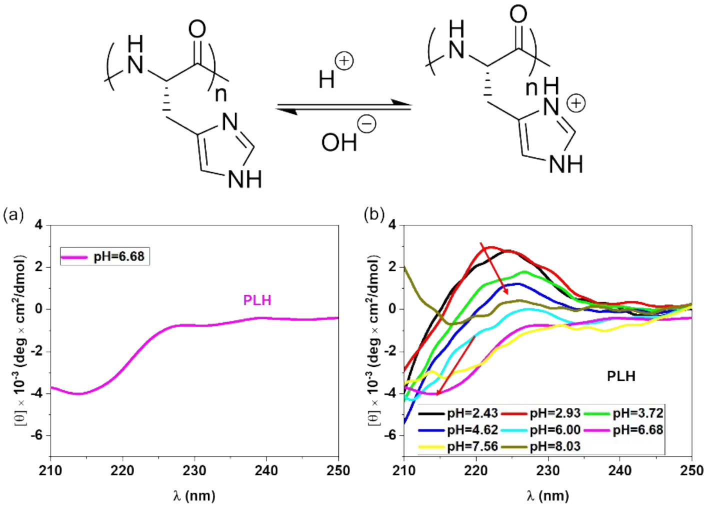

Figure S22. CD spectra of PLH in aqueous solution: (a) β-sheet conformation at pH = 6.68; (b) conformational transition at pH 2.43-8.03. Concentration = 0.025 mg/mL.

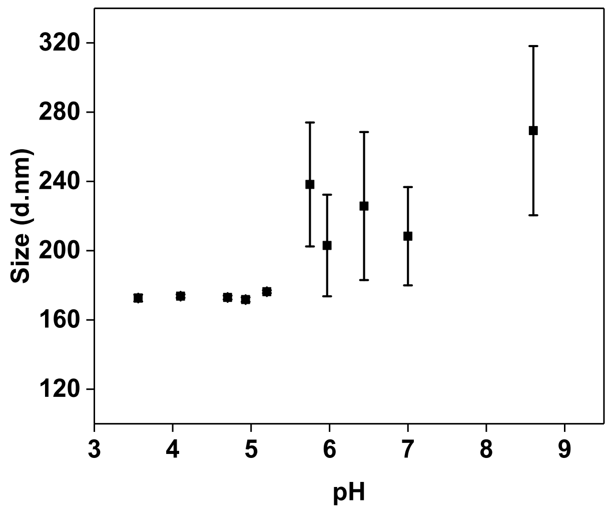

Figure S23. Diameters of PPLG-EG$_2$ nanoparticles in aqueous solutions at different pH values (n = 3). The tested concentration was 1 mg/mL.

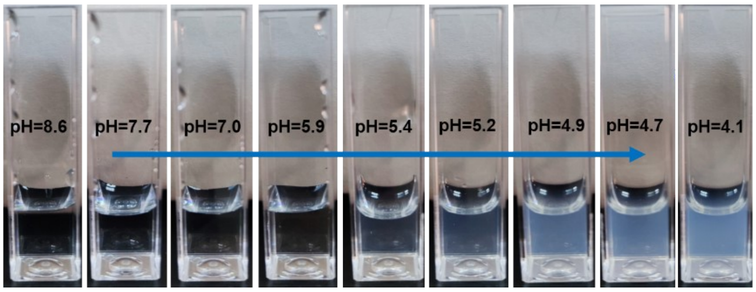

Figure S24. Images of PPLG-EG$_2$ nanoparticles in aqueous solutions at different pH. The tested concentration was 1 mg/mL.

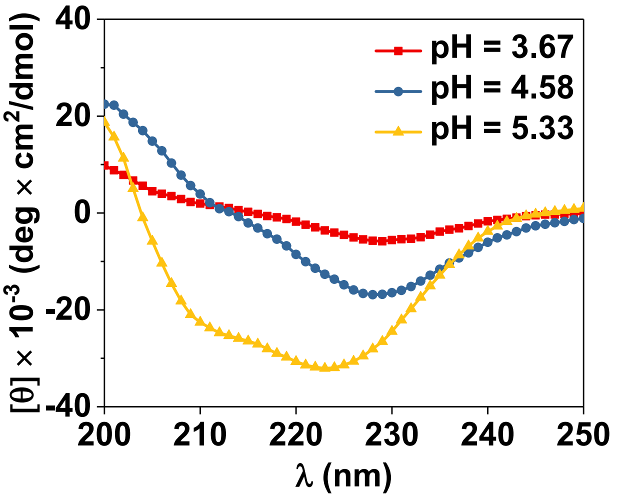

Figure S25. CD spectra of PPLG-Hex in aqueous solution at different pH. Concentration = 0.025 mg/mL.

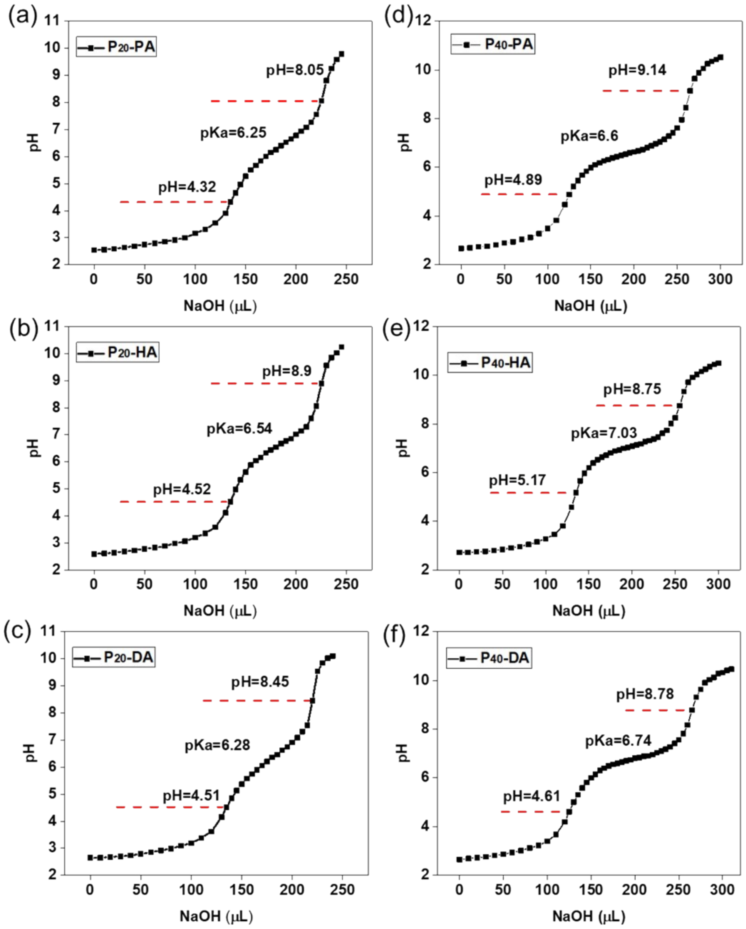

Figure S26. pH titrations of SAi-polypeptides adjusted using 0.1 M NaOH solution: (a) P_{20}-PA, (b) P_{20}-HA, (c) P_{20}-DA, (d) P_{40}-PA, (e) P_{40}-HA, and (f) P_{40}-DA. The polymer concentration was 1 mg/mL.

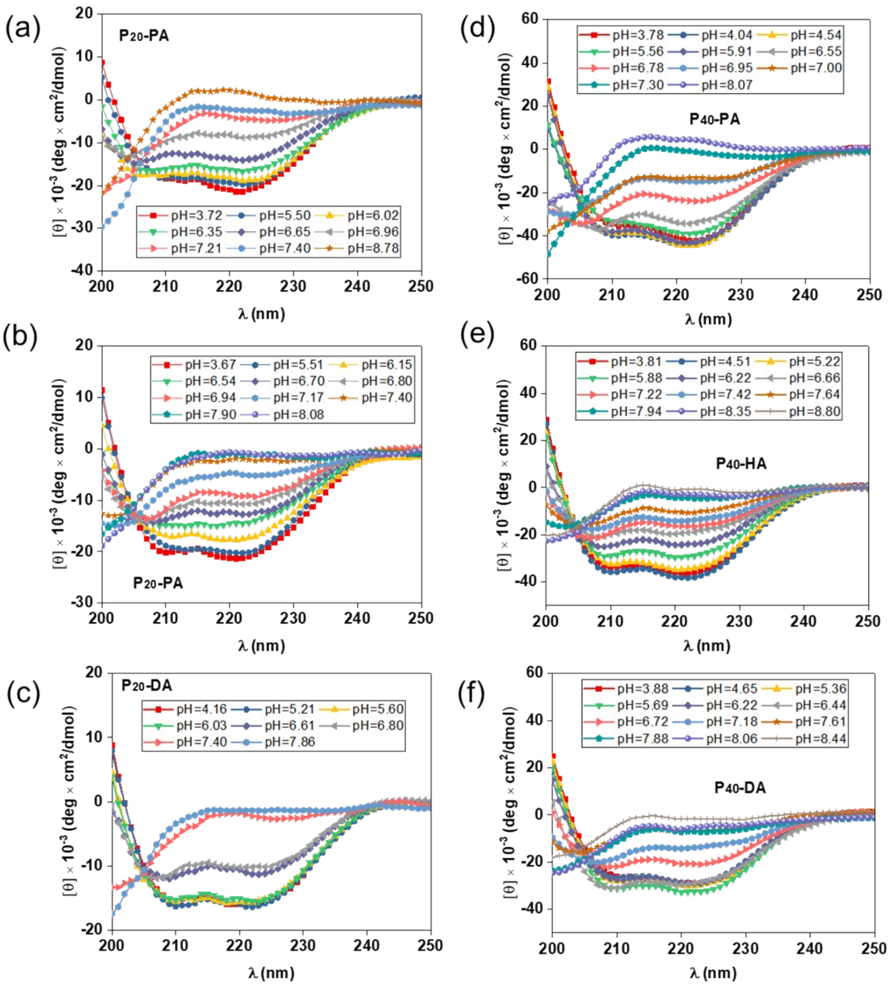

Figure S27. CD spectra of polypeptides in aqueous solution: (a) P_{20}-PA, (b) P_{20}-HA, (c) P_{20}-DA, (d) P_{40}-PA, (e) P_{40}-HA, and (f) P_{40}-DA. Concentration = 0.025 mg/mL.

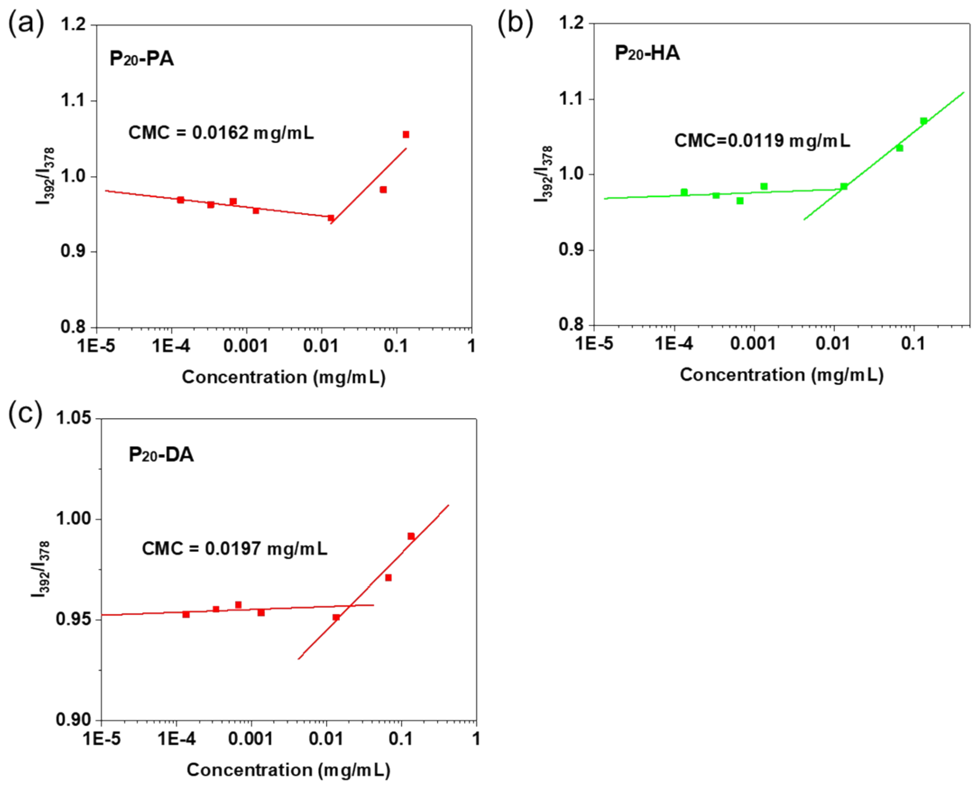

Figure S28. The dependence of excitation fluorescence intensity ratio (I_{392}/I_{378}) on the logarithmic concentration of blank micelles: (a) P_{20}-PA, (b) P_{20}-HA, (c) P_{20}-DA. The CMC values of polypeptides were calculated from the intersection point

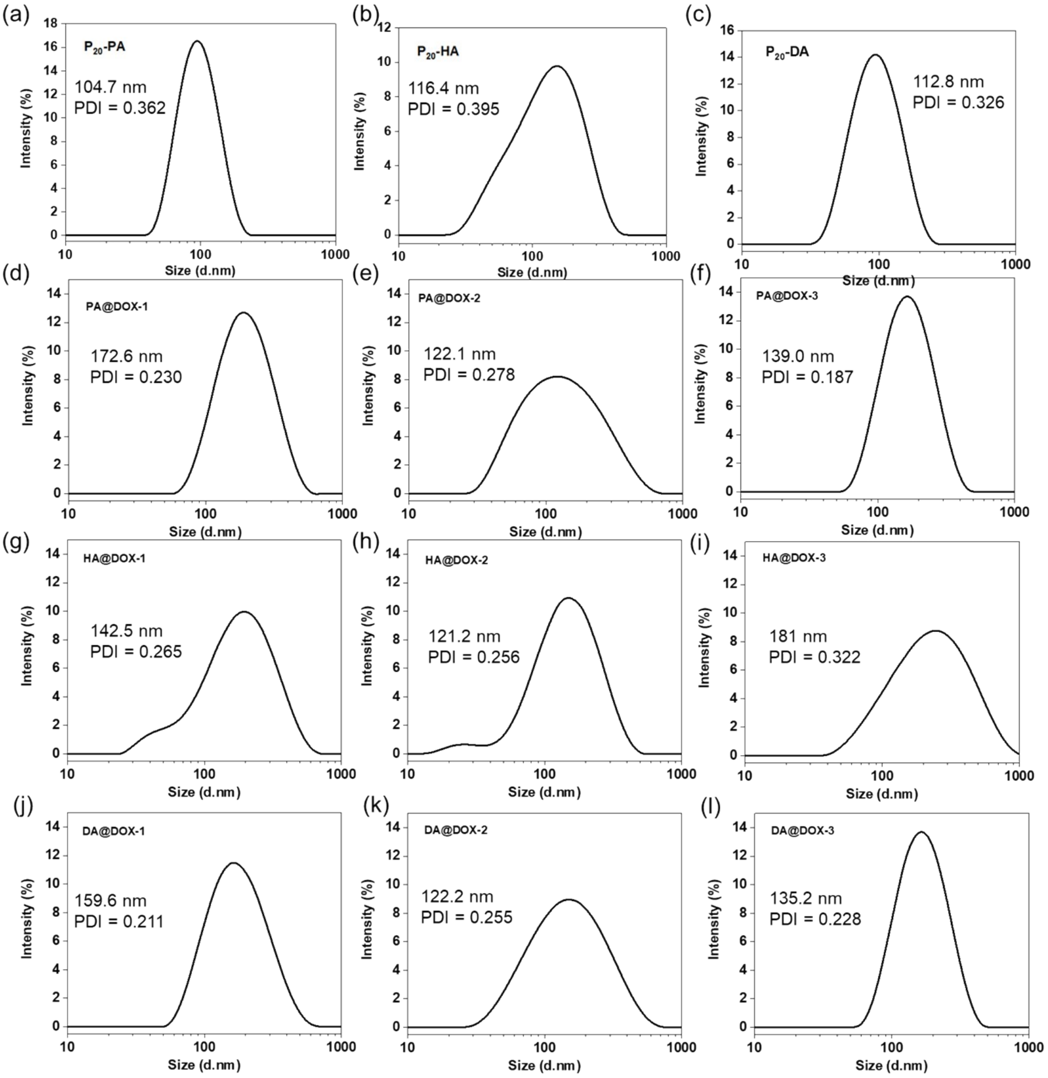

Figure S29. Intensity size distributions of blank micelles: (a) P_{20}-PA, (b) P_{20}-HA, (c) P_{20}-DA. Intensity size distribution of drug-loaded micelles: (d) PA@DOX-1, (e) PA@DOX-2, (f) PA@DOX-e, (g) HA@DOX-1, (h) HA@DOX-2, (i) HA@DOX-3, (j) DA@DOX-1, (k) DA@DOX-2, (l) DA@DOX-3.

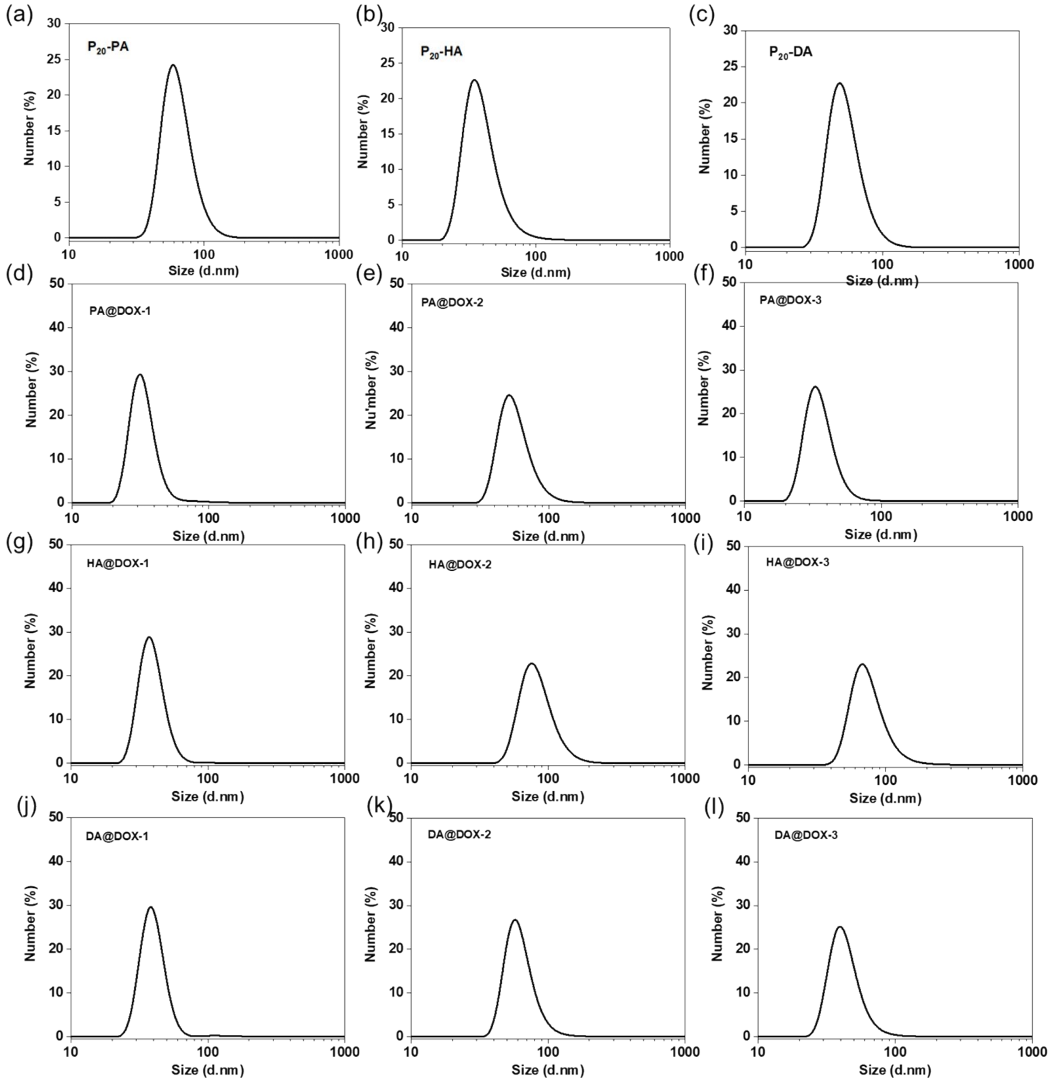

Figure S30. Number size distribution of blank micelles: (a) P_{20}-PA, (b) P_{20}-HA, (c) P_{20}-DA. Number size distribution of drug-loaded micelles: (d) PA@DOX-1, (e) PA@DOX-2, (f) PA@DOX-e, (g) HA@DOX-1, (h) HA@DOX-2, (i) HA@DOX-3, (j) DA@DOX-1, (k) DA@DOX-2, (l) DA@DOX-3.

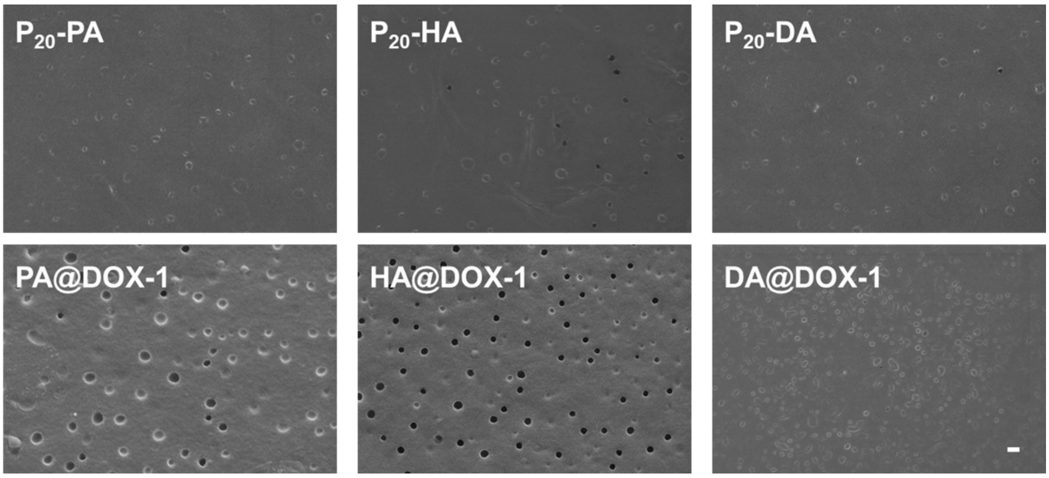

Figure S31. SEM images of blank micelles and DOX-NPs. Scale bar: 300 nm.

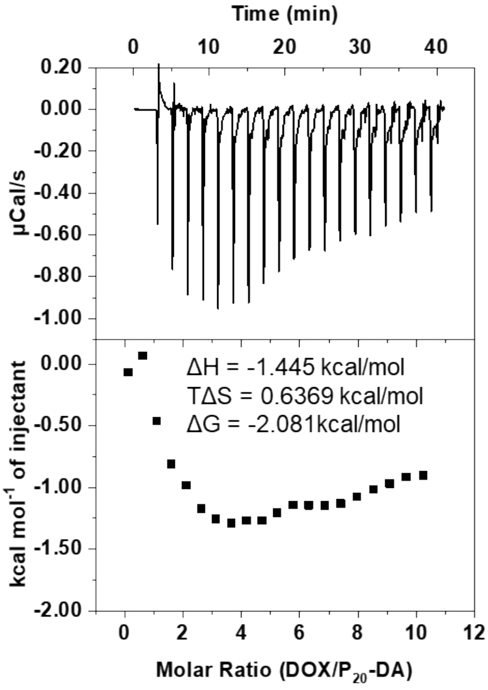

Figure S32. ITC data obtained from titration 3.3 mM DOX in P_{20}-DA in PBS solution.

Data analysis was performed using the software Microcal LLC ITC.

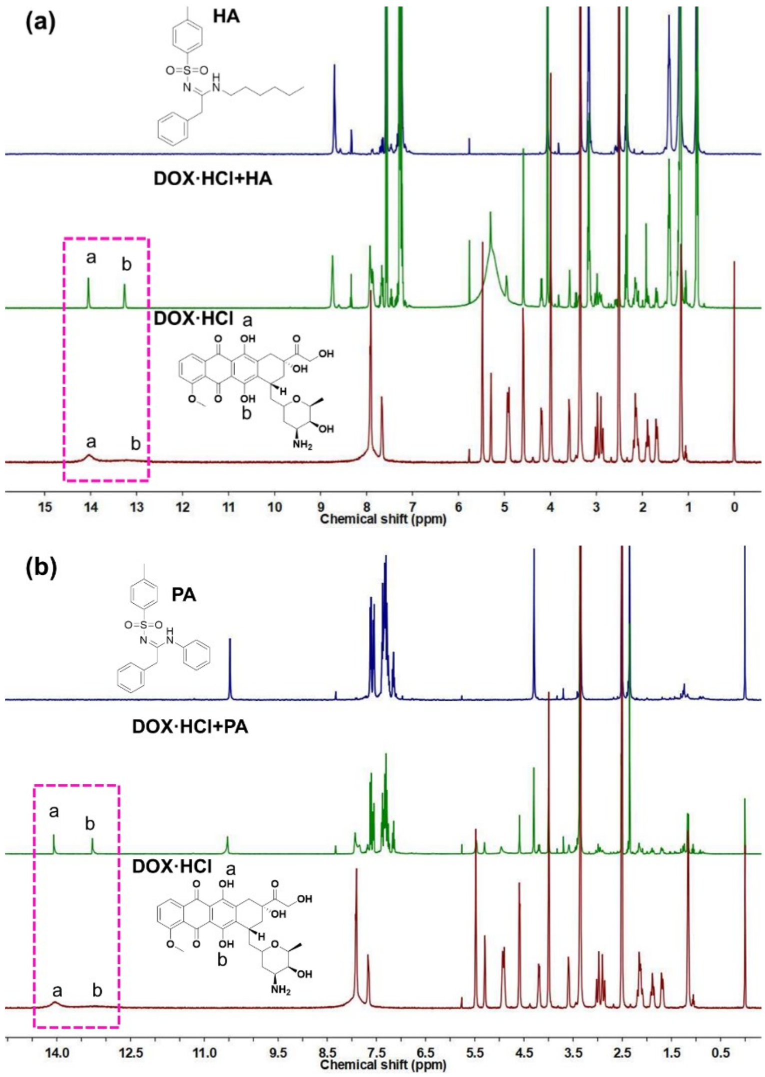

Figure S33. ^{1}H NMR spectra of mixture of SAi small molecules and DOX·HCl in DMSO-d_6: (a) compound HA and DOX·HCl, (b) compound PA and DOX·HCl.

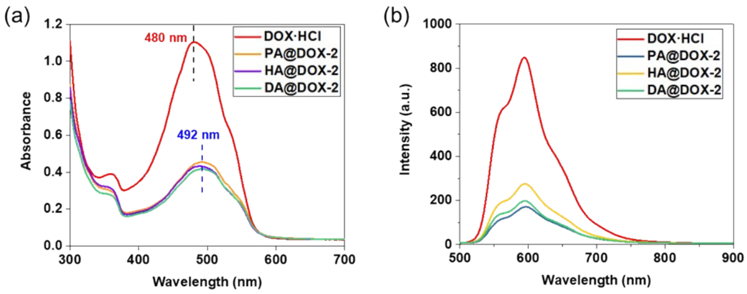

Figure S34. (a) UV-vis spectra of micelles in aqueous solution. The concentration of DOX was 86 μM. (b) Fluorescence spectra of micelles in aqueous solution at 480 nm excitation wavelength. The concentration of DOX was 86 μM.

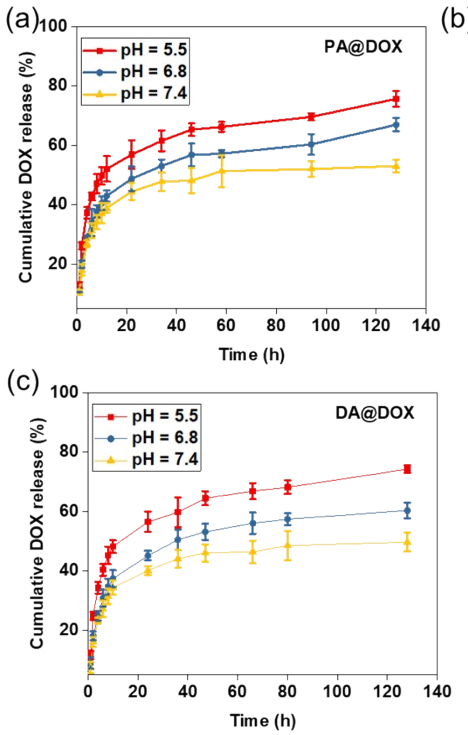

Figure S35. The release profiles of DOX from the DOX-NPs: (a) PA@DOX-2, (b) HA@DOX-2, and (c) DA@DOX-2.

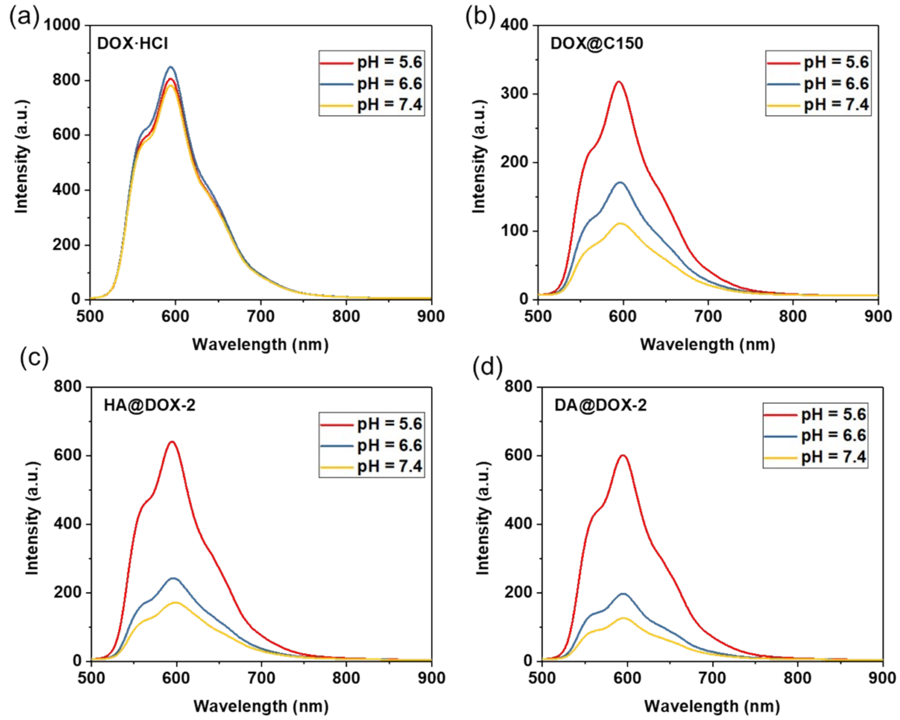

Figure S36. Fluorescence spectra of DOX·HCl and micelles in aqueous solution at 480 nm excitation wavelength: (a) DOX·HCl, (b) PA@DOX-2, (c) HA@DOX-2, and (d) DA@DOX-2. The concentration of DOX was 86 μM.

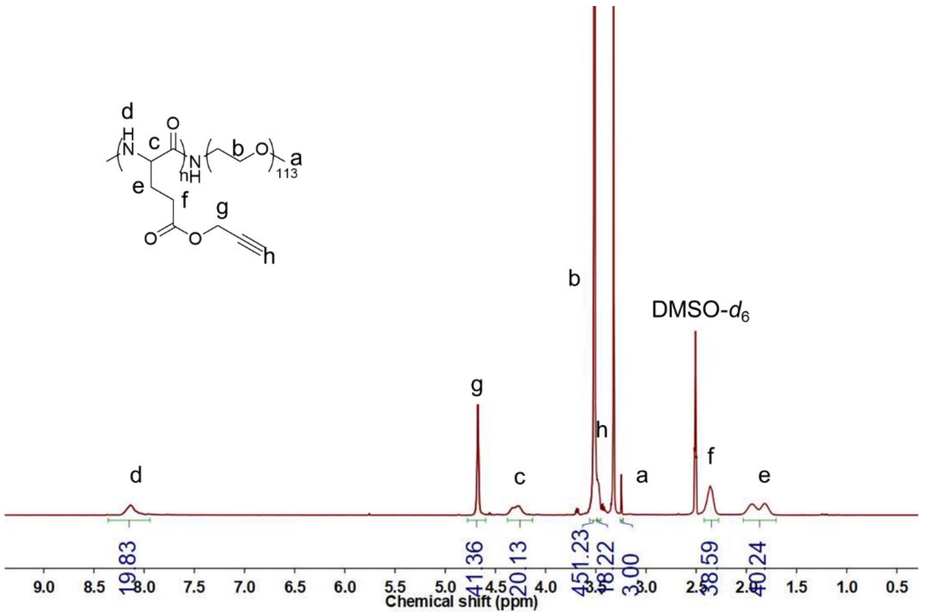

Figure S37 ^{1}H NMR spectrum of PEG-PPDLG$_{20}$ in DMSO-d_6.

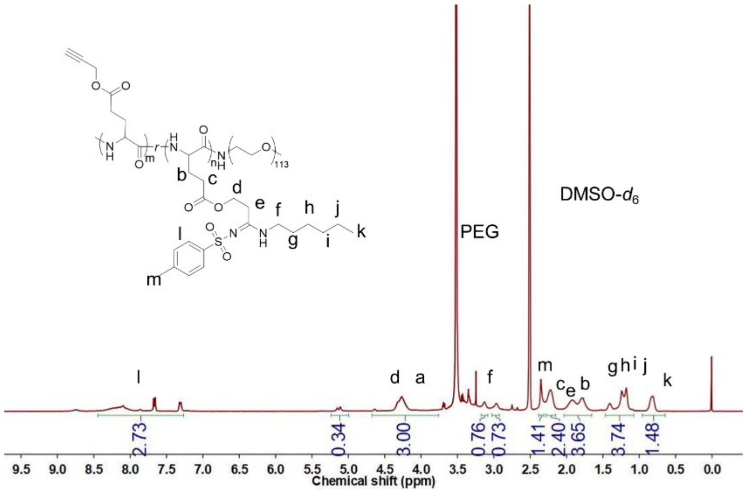

Figure S38 ^{1}H NMR spectrum of DL-P$_{20}$-HA in DMSO-d_6.

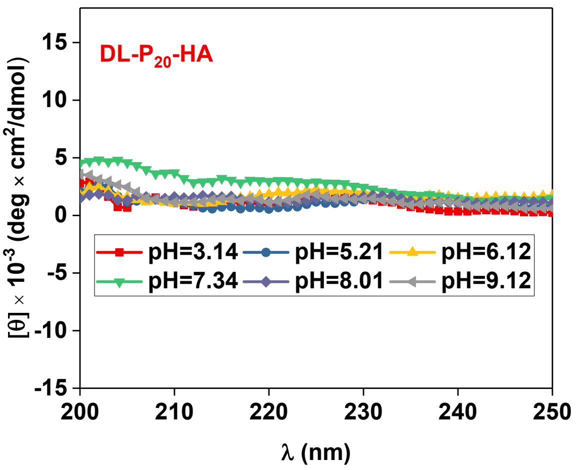

Figure S39. CD spectra of DL-P$_{20}$-HA in aqueous solution. Concentration = 0.025

mg/mL.

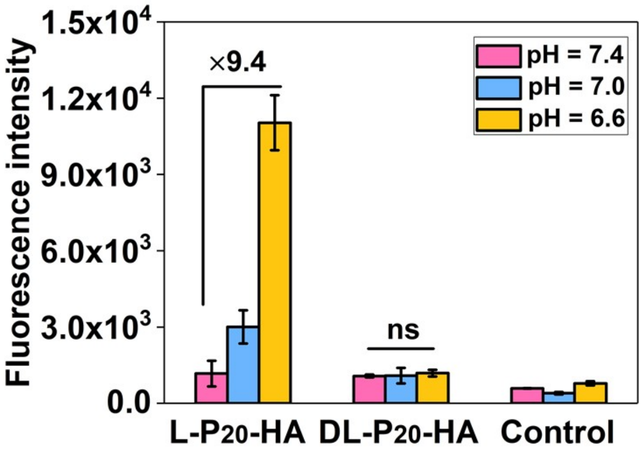

Figure S40. FITC-Tris uptake level of Hela cells following co-incubation with DL-P_{20}-HA and L-P_{20}-HA. The control means cells treated without polypeptides. The biological parallel experiments were carried out for three times (n = 3). The ns means not statistically significant.

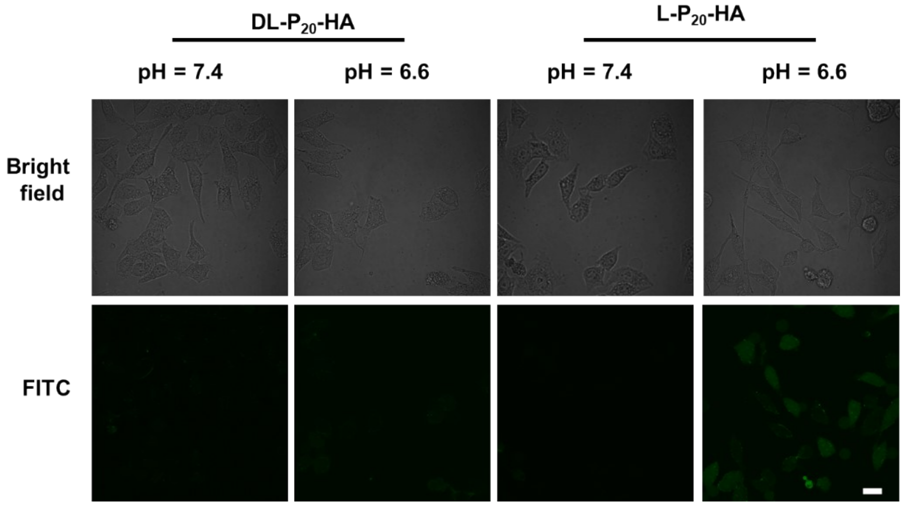

Figure S41. CLSM images showing FITC-tris uptake level of Hela cells treated with DL-P_{20}-HA or L-P_{20}-HA for 4 h at pH = 6.6 and 7.4. Scale bar = 20 μm.

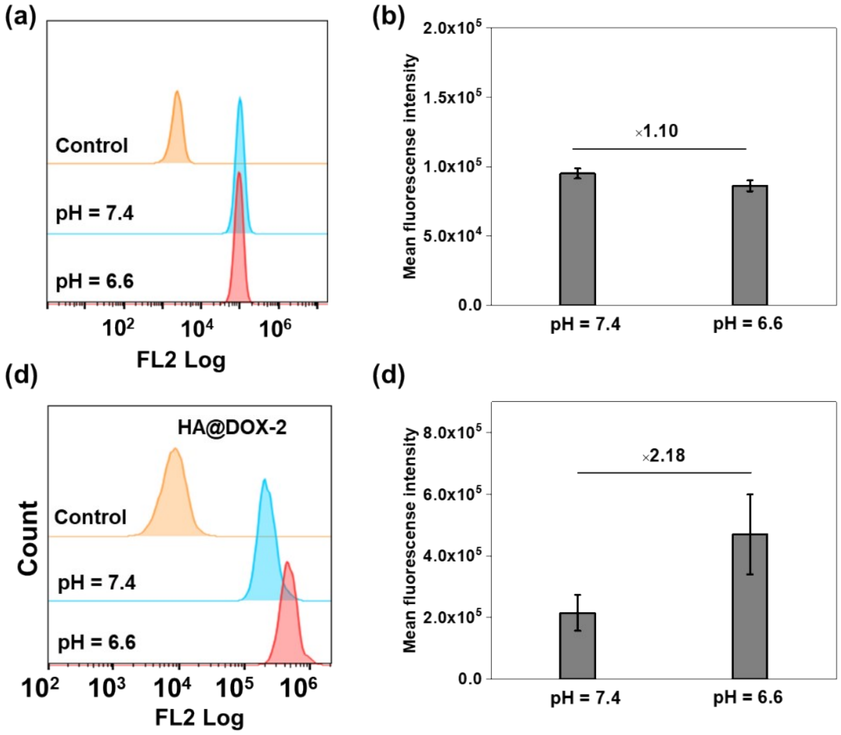

Figure S42. FCM histogram profiles (a) and corresponding mean fluorescence intensity (b) of Hela cells treated with DOX-NPs (prepared from DL-P_{20}-HA) for 6 h. FCM histogram profiles (c) and corresponding mean fluorescence intensity (d) of Hela cells treated with DOX-NPs (prepared from L-P_{20}-HA) for 6 h. n = 3.

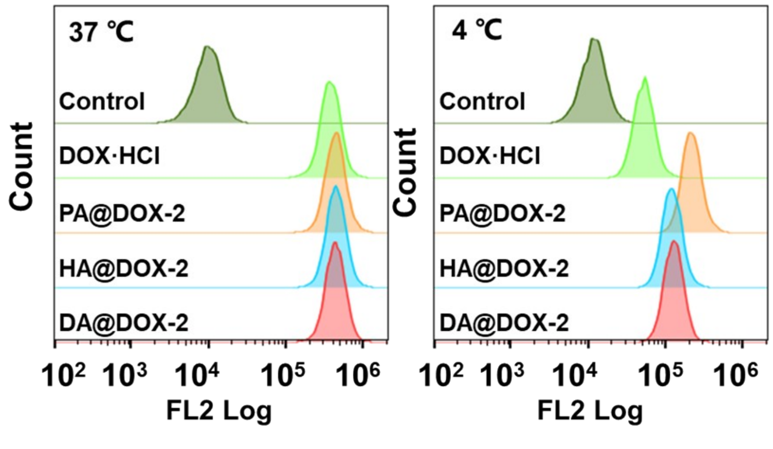

Figure S43. FCM histogram profiles of Hela cells treated with DOX-NPs at 4 °C and 37 °C.

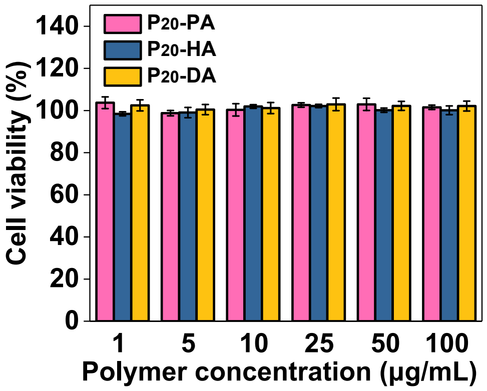

Figure S44. Cell viability of the cells incubated with blank micelles at pH =7.6 (n = 3).

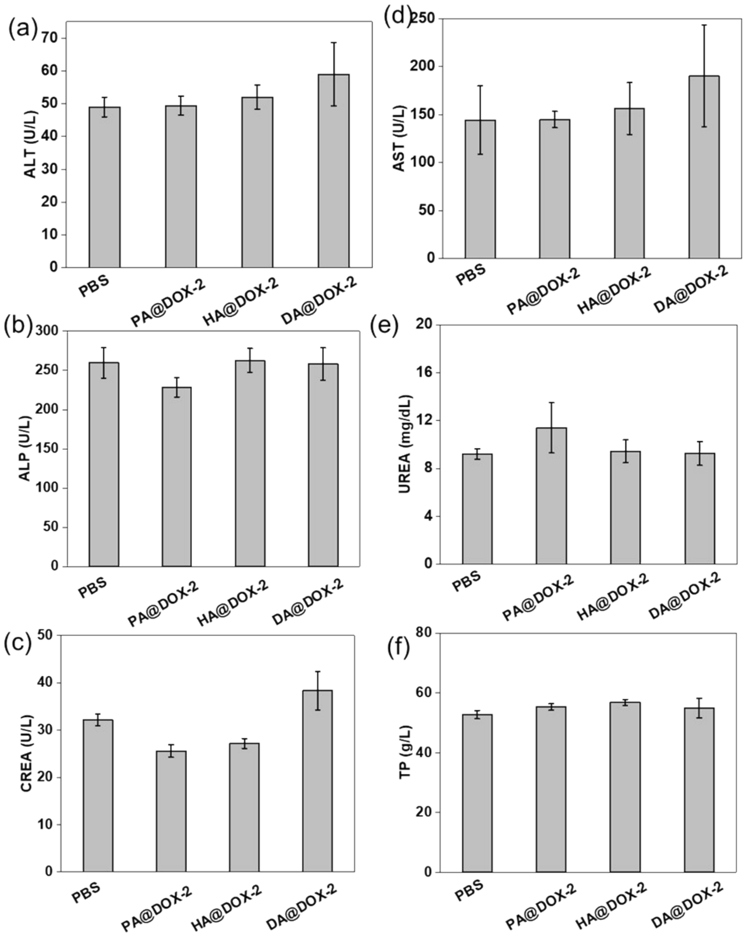

Figure S45. Blood biochemistry indexes in mice after different treatments.

Table S2. Results of the hematology tests from all study groups

Contents	PBS	PA@DOX-2	HA@DOX-2	DA@DOX-2
WBC	4.9±0.67	4.3±1.1	3.5±0.4	4.8±0.9
Neu (×10^9/L)	1.0±0.03	1.1±0.7	0.8±0.2	1.1±0.2
Neu (%)	21.3±3.2	25.2±5.6	22.2±2.3	22.8±1.0
Lym (×10^9/L)	3.8±0.7	3.0±0.9	2.6±0.4	3.5±0.6
Lym (%)	76.02±3.7	69.9±12.7	75.0±2.7	73.7±1.8
Mon(×10^9/L)	0.1±0.0	0.1±0.0	0.1±0.0	0.1±0.0
Mon (%)	1.0±0.3	1.1±0.4	1.3±0.3	4.8±0.9
Eos (×10^9/L)	0.1±0.0	0.1±0.0	0.1±0.0	0.8±0.4
Eos (%)	1.2±0.6	1.2±0.4	0.8±0.4	0.8±0.3
Bas (×10^9/L)	0.1±0.0	0.1±0.0	0.1±0.0	0.1±0.0
Bas (%)	0.3±0.2	0.5±0.1	0.7±0.1	0.6±0.1
RBC(×10^{12}/L)	8.7±0.2	7.8±1.6	8.5±0.1	9.0±0.5
HGB (g/L)	142.3±5.5	132.2±25	141.0±4	150±5.6
HCT (%)	42.9±7.8	38.9±7.5	42.3±0.9	44.7±2.0
MCV (fL)	49.1±0.9	49.9±0.7	49.5±0.5	49.7±0.5
MCH (pg)	16.3±0.2	16.9±0.3	16.5±0.2	16.8±0.3
MCHC (g/L)	331.6±3.5	339±2.6	335±3.6	337±7.5
RDW-CV (%)	14.2±0.6	14.6±0.5	14.8±0.9	14.3±0.3
RDW-SD (fL)	30.2±1.0	31.5±0.9	32.0±1.8	30.9±0.6
PLT (×10^9/L)	849.3±206.4	522±216	1029±60.5	583±253
MPV (fL)	5.2±0.1	5.4±0.2	5.2±0.2	5.1±0.0
PDW	15.8±0.1	16.2±1.5	15.5±0.3	15.3±0.2
PCT (%)	0.4±0.1	0.1±0.1	0.5±0.1	0.2±0.0

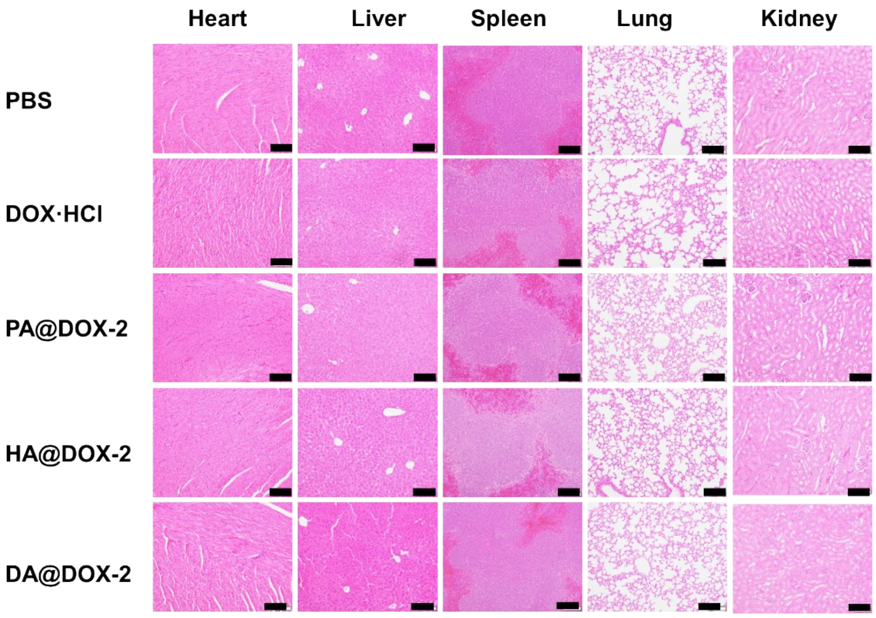

Figure S46. Histological staining (H&E) of tissue sections from mice to monitor the histological changes in heart, liver, spleen, lung, and kidney in each group after 14 days of treatment. Scale bar: 1 mm.

Reference

[1] C. S. Xiao, C. W. Zhao, P. He, Z. H. Tang, X. S. Chen, X. B. Jing, *Macromolecular Rapid Communications*, **2010**, *31*, 991-997.

[2] X. Xu, R. M. Liang, Y. B. Zheng, Y. Qiu, N. Zheng, *Biomacromolecules*, **2023**, *24*, 3115-3126.

[3] I. Bae, H. Han, S. Chang, *Journal of the American Chemical Society*, **2005**, *127*, 2038-2039.

[4] X. P. Tian, C. Y. Wang, X. H. Jin, M. Li, F. W. Wang, W. J. Huang, J. P. Yun, R. H. Xu, Q. Q. Cai, D. Xie, *Theranostics*, **2019**, *9*, 1965-1979.

[5] J. W. Wojtkowiak, J. M. Rothberg, V. Kumar, K. J. Schramm, E. Haller, J. B. Proemsey, M. C. Lloyd, B. F. Sloane, R. J. Gillies, *Cancer Research*, **2012**, *72*, 3938-3947.

[6] S. X. Lv, Y. C. Wu, K. M. Cai, H. He, Y. J. Li, M. Lan, X. S. Chen, J. J. Cheng, L. C. Yin, *Journal of the American Chemical Society*, **2018**, *140*, 1235-1238.
